# Supplementary material for: Quantification of human contribution to soil moisture-based terrestrial aridity
Source: Nat Commun. 2022 Nov 11;13:6848. doi: 10.1038/s41467-022-34071-5 (PMC9652356; doi:10.1038/s41467-022-34071-5)
Supplement: Supplementary file 1 — Supplementary information [file 41467_2022_34071_MOESM1_ESM.docx]

Quantification of human contribution to soil moisture–based terrestrial aridity

Yaoping Wang^1,2**^, Jiafu Mao^2,*,**^, Forrest M. Hoffman^3^, Céline J. W. Bonfils^4^, Hervé Douville^5^, Mingzhou Jin^1,6^, Peter E. Thornton^2^, Daniel M. Ricciuto^2^, Xiaoying Shi^2^, Haishan Chen^7^, Stan D. Wullschleger^2^, Shilong Piao^8^, and Yongjiu Dai^9^

*^1^ Institute for a Secure and Sustainable Environment, University of Tennessee, Knoxville, TN, USA*

*^2^ Environmental Sciences Division and Climate Change Science Institute, Oak Ridge National Laboratory, Oak Ridge, TN, USA*

*^3^ Computational Sciences and Engineering Division and Climate Change Science Institute, Oak Ridge National Laboratory, Oak Ridge, TN, USA*

*^4^ Program for Climate Model Diagnosis and Intercomparison, Atmospheric, Earth, & Energy Division, Lawrence Livermore National Laboratory, Livermore, CA, USA*

^5^ *Centre National de Recherches Météorologiques, CNRM/GMGEC/AMACS, Université de Toulouse, Météo-France, CNRS, Toulouse Cedex 01, France*

*^6^ Department of Industrial and Systems Engineering, University of Tennessee, Knoxville, TN, USA*

*^7^ Key Laboratory of Meteorological Disaster, Ministry of Education/Joint International Research Laboratory of Climate and Environment Change/Collaborative Innovation Center on Forecast and Evaluation of Meteorological Disasters, Nanjing University of Information Science & Technology, Nanjing, China*

*^8^* *Sino-French Institute for Earth System Science, College of Urban and Environmental Sciences, Peking University, Beijing, China*

^9^ *School of Atmospheric Sciences, Sun Yat-sen University, Guangzhou, China*

***^*^***Jiafu Mao: Environmental Sciences Division and Climate Change Science Institute, Oak Ridge National Laboratory, Oak Ridge, TN 37830; +1 865-576-7815; [maoj@ornl.gov](mailto:maoj@ornl.gov)

^**^These authors contributed equally to this work. ^[[1]](#footnote-2)^†

**Supplementary information**

**Table of Contents**

[Supplementary Methods 3](#_Toc115801991)

[1. Calculation of the Standardized Soil Moisture Index 3](#_Toc115801992)

[1.1. Source and preprocessing of soil moisture data 3](#_Toc115801993)

[1.2. Calculation of the SSI 3](#_Toc115801994)

[2. D&A analysis 4](#_Toc115801995)

[2.1. Pre-processing 4](#_Toc115801996)

[2.2. Fingerprint 4](#_Toc115801997)

[2.3. Noise 5](#_Toc115801998)

[2.4. Signal 5](#_Toc115801999)

[2.5. Signal-to-noise ratio, significance, and consistency 6](#_Toc115802000)

[3. Sensitivity analysis on the results of D&A 6](#_Toc115802001)

[4. Physical justification for the emergent constraint relationship between modeled historical and future S/N ratios 7](#_Toc115802002)

[5. Relationship between the S/N ratio and the trends in the SSI under the ALL forcing 7](#_Toc115802003)

[Supplementary Figures 10](#_Toc115802004)

[Supplementary Tables 23](#_Toc115802005)

[Supplementary References 32](#_Toc115802006)

# Supplementary Methods

# Calculation of the Standardized Soil Moisture Index

## Source and preprocessing of soil moisture data

Standardized Soil Moisture Index (SSI) was calculated from seven previously developed merged soil moisture products, which span 1970–2016 and cover the globe at 0.5° resolution and the soil layers 0–10, 10–30, 30–50, and 50–100 cm^1^. Among these products, three were independent of the Coupled Model Intercomparison Project Phase 5 or 6 (CMIP5 or CMIP6) Earth system models (ESMs) (Mean-ORS, OLC-ORS, EC-ORS), and four depended on the ESMs (EC-CMIP5, EC-CMIP6, EC-CMIP5+6, EC-ALL) (Table 1). The unweighted average SSI of the three CMIP‑independent products were used as the pseudo-observation (Mean NonCMIP) for the detection and attribution (D&A) analysis in the main text. The unweighted average SSI of all seven products (Mean Products) were used for sensitivity analysis (Section 3). The original soil moisture values were linearly aggregated to 0–10 and 0–100 cm proportional to the original soil layer thickness. Regions with sparse vegetation (i.e., Normalized Difference Vegetation Index [NDVI]) <0.125) were masked, based on the Advanced Very High Resolution Radiometer NDVI3g data set,^2^ because soil moisture values in these arid regions may be subject to influence from random noise^3^.

The ESM data for the D&A analysis were from the “mrsol” variable of the CMIP6 Diagnostic, Evaluation and Characterization of Klima (DECK) and the Detection and Attribution Model Intercomparison Project (DAMIP) simulations^4,5^, and were downloaded from the Earth System Grid Federation^6^. Table 2 lists the simulations that were used to represent different external forcings, and Table 3 lists the ensemble members in each simulation. The ALL simulations were obtained by concatenating the historical integrations (which typically ends in 2014) and the Shared Socioeconomic Pathway 5-8.5 (SSP5-8.5) future simulations (which start in 2015) to cover the two years in pseudo‑observation (2015 and 2016) that are not in the historical integrations, following previous D&A studies^7–11^. The ANT (forced by combined anthropogenic factors only) signals were estimated by subtracting the NAT simulations from the ALL simulations, and the GHGAER (forced by both greenhouse gases and anthropogenic aerosols) signals were derived by adding the GHG and AER simulations (Table 2). Such calculations to obtain the ANT and GHGAER signals assume the linear additivity of the responses to forcing agents, following past studies^12,13^. All the ESMs were bilinearly interpolated to 0.5° resolution and linearly aggregated to 0–10 and 0–100 cm proportional to the soil layer thickness before calculating the SSI. Regions with sparse vegetation were masked like the pseudo-observation.

## Calculation of the SSI

The SSI calculation followed a procedure analogous to the Standardized Precipitation Index (SPI) and involved three steps^14–16^. First, for each month of the year, a predetermined statistical distribution function was fitted to the averaged soil moisture values of the k consecutive months that end in the current month (k = 1, 3, or 6). Then, the averaged soil moisture values were converted to percentiles using the cumulative distribution function of the fitted statistical distribution. Finally, the percentiles were converted to z-scores on the standard Gaussian distribution, i.e., the SSI. The whole procedure was implemented individually for each 0.5° × 0.5° grid, month, merged soil moisture product (Table 1), and CMIP6 ESM (Table 3). Throughout the text, we always referred to the SSI values by the last month that the averaging periods ended in. For example, the 3-month SSI of a February would be based on the average soil moisture of the previous year’s December and this year’s January–February, and the 6-month SSI of a June would be based on the averaged soil moisture of this year’s January–June. The same definition applied when the SSI of multiple months were averaged (e.g., the 3-month SSI of DJF means the average value of the 3-month SSI of December, January, and February).

The predetermined statistical distribution function was chosen to be Gaussian mixture model (GMM)^17^ in the main text, and the Weibull distribution^18^ in sensitivity analysis (Section 3). These distribution were selected based on past literature^17,18^ and goodness-of-fit on the SSI. The GMM was used for the main text because of its higher log-likelihood of fit than the Weibull distribution (Figure 1)**.** For the pseudo-observation, the GMM or Weibull distributions were fitted on the k-month average soil moisture values during 1970–2014, and used to obtain the z-scores during 1970–2016. For each CMIP6 ESM, the GMM or Weibull distributions were fitted on the k-month average soil moisture values during 1970–2014 pooled from all the available historical ensemble members of the ESM (Table 3), and used to obtain the z-scores for all the simulations of the ESM regardless of the external forcing (piControl, historical, SSP5-8.5, hist-GHG, hist-aer, or hist-nat) or ensemble member. The reference period 1970–2014 was chosen because it is the longest period of overlap between the historical simulations (1850–2014) and the merged soil moisture products (1970–2016). For the CMIP6 ESMs, using all the available ensemble members to fit the statistical distributions provided as large a data pool as possible for robust fitting.

# D&A analysis

## Pre-processing

We aggregated the 0.5° gridded SSI of the pseudo-observation and the CMIP6 simulations to 5° zonal averages, weighted by the area of each 0.5° grid cell, to reduce small-scale variability and enhance S/N ratio, following previous studies on the D&A of hydrological variables^11,12,19–21^.

## Fingerprint

The fingerprint, $F_{f_{1}}\left( \theta,m \right)$, characterizes the spatial pattern of the response of the zonal mean SSI to the external forcing f_1_, and is a function of latitude θ (25 latitudes, centered around 50°S, 45°S, 40°S, …, 70°N) and month of the year, m (12 months). The calculation of the fingerprints had three steps:

1. The CMIP6 simulations under the f_1_ forcing were first averaged over the ensemble members of each model and then averaged over the models to derive a weighted ensemble average zonal mean SSI, $S_{f_{1}}\left( \theta,m,t \right)$, where t denotes the year. The weighted averaging procedure ensured that the models with more ensemble members would not disproportionately affect the fingerprint.
2. The fingerprint was calculated for each individual month m as the leading empirical orthogonal function (EOF) of $S_{f_{1}}\left( \theta,m,t \right)\cdot a\left( \theta\right)$ over the time periods 1971–2100 and 1971–2020 for the ALL simulations, and over the time period 1971–2020 for the GHG and AER simulations). The scaling term $a\left( \theta\right)=\sqrt{A\left( \theta\right)}/\left[ \frac{1}{25}\sum_{\theta} \sqrt{A\left( \theta\right)} \right]$, where $A\left( \theta\right)$ is the land area of the 5° latitudinal band centered around $\theta$, ensured that the latitudes with smaller land area received less weight in the calculation of the EOF.
3. For better visualization, the signs of all the EOF fingerprints were flipped to ensure that their corresponding principal components (PCs) had positive Spearman trends over the period of calculation (i.e., 1971–2100 and 1971–2020 for ALL, 1971–2020 for GHG and AER). Since the EOFs and their PCs are the eigenvalues and PCs from the eigenvalue decomposition of the covariance matrix of the input data^22^, they can be simultaneously multiplied by −1 without affecting the mathematical validity.

We calculated the ALL fingerprint (i.e., f_1_ = ALL) of each month using the ALL simulations over 1971–2100 (Table 2). The ALL fingerprints were used for D&A throughout the text. We also calculated the alternative ALL-2 fingerprints using the ALL simulations over 1971–2020, and the GHG and AER fingerprints (i.e., f_1_ = GHG and AER, respectively) using the GHG and AER simulations over 1971–2020 (Table 2). The ALL-2, GHG, and AER fingerprints were only used in the main text Fig. 2 for comparison purpose.

## Noise

The noise term characterizes the degree to which natural internal variability can resemble the spatial pattern of the SSI fingerprint by chance. We calculated the noise term using CMIP6 piControl simulations (Table 3). For each model ensemble member, we only used the last 200 years of the simulation to prevent the models with longer piControl simulations from dominating the noise estimation^9^. We first converted the zonally averaged SSIs to anomalies relative to the 200-year climatology of each model ensemble member and month, following previous practice^9^. Then, we concatenated all the anomalies together to create two 3,800-year (19 members × 200 years) space-time series for the 0–10 and 0–100 cm SSI, respectively.

We projected the concatenated space-time series, $C\left( \theta,m,t \right)$, on the ALL fingerprint, $F_{f_{1}}\left( \theta,m \right)$ (f_1_ = ALL), weighted by the square root of the land area of each 5° latitudinal band to obtain a time series $N_{f_{1}}\left( m,t \right)$, following Eq. (1).

|  | $N_{f_{1}}\left( m,t \right)=\sum_{\theta} C\left( \theta,m,t \right)\cdot F_{f_{1}}\left( \theta,m \right)\cdot a\left( \theta\right) t=1,\ldots,3,800$ | (1) |
| --- | --- | --- |

For any given month m and time window length of L years, all the possible L-year trends, $T_{f_{1}}\left( m,t \right)$, were calculated by applying linear least squares on all the possible L-year moving window segments in the series $N_{f_{1}}\left( m,t \right)$. Those L-year trends were called “unforced trends”. Then, we calculated a single standard deviation, $\hat{\sigma}_{f_{1}}\left( m,L \right)$, over all the L-year trends $T_{f_{1}}\left( m,t \right)$, t = 1,…,3,800–L. This standard deviation was called the “noise” (N). We also calculated the 95% confidence interval (CI) for the unforced trends as $-1.96\times\hat{\sigma}_{f_{1}}\left( m,L \right)$ to $1.96\times\hat{\sigma}_{f_{1}}\left( m,L \right)$ using the Gaussian assumption, following previous D&A studies on hydrologic variables^7,11^.

## Signal

The signal term characterizes the degree of similarity between the fingerprint pattern, and the observed SSI patterns or the simulated SSI patterns under a specific forcing. To calculate the pseudo-observed signal, we first projected the zonally averaged SSI of the pseudo-observation, $O\left( \theta,m,t \right)$, onto the ALL fingerprint following Eq. (2).

|  | $Z_{f_{1}o}\left( m,t \right)=\sum_{\theta} O\left( \theta,m,t \right)\cdot F_{f_{1}}\left( \theta,m \right)\cdot a\left( \theta\right) t=1971,\ldots, 2016$ | (2) |
| --- | --- | --- |

, where the subscript f_1_ = ALL and o denotes pseudo-observation. Then, the linear least squares trend $G_{f_{1}o}\left( t_{0},m,L \right)$ over the time series $Z_{f_{1}o}\left( m,t \right)$ was calculated as the pseudo-observed signal (S) on the ALL fingerprint, for a given month m, starting year t_0_, and time window of L years. When a signal was detectable in the D&A analysis, it should be understood as being detectable over the time period specified by t_0_ and L. The time periods of the signals and signal-to-noise (S/N) ratios varied in this study and were specified where the signals and S/N ratios were described.

Similarly, to calculate the signals of the simulations under a specific forcing f_2_ (f_2_ = ALL, ANT, GHG, AER, GHGAER, or NAT), we first projected the zonally averaged SSIs of each model (d) and ensemble member (i) under the f_2_ forcing, $S_{f_{2}\mathrm{di}}\left( \theta,m,t \right)$, onto the ALL fingerprint following Eq. (3). The models and ensemble members used for each forcing were shown in Table 3.

|  | $Z_{f_{2}f_{1}\mathrm{di}}\left( m,t \right)=\sum_{\theta} S_{f_{2}\mathrm{di}}\left( \theta,m,t \right)\cdot F_{f_{1}}\left( \theta,m \right)\cdot a\left( \theta\right) t=1971,\ldots, 2100 or 2020$ | (3) |
| --- | --- | --- |

Then, for a given month m, starting year t_0_, and time window of L years, we calculated the linear least squares trends $G_{f_{1}o}\left( t_{0},m,L \right)$ for the time series $Z_{f_{2}f_{1}\mathrm{di}}\left( m,t \right)$, and called the trend $G_{f_{2}f_{1}\mathrm{di}}\left( t_{0},m,L \right)$ the simulated forced signals for each model (d), ensemble member (i) and set of forcings f_2_ (sometimes “simulated signals” or “f_2_-forced signals” for brevity).

## Detection

We followed previous studies ^7,8,12^ in considering the pseudo-observed signal to be detectable at the 95% confidence level when the signal was outside the two-sided 95% CI of the distribution of the unforced trends. As mentioned in Sect. 2.3, this 95% CI of the unforced trends was estimated as $-1.96\times\hat{\sigma}_{f_{1}}\left( m,L \right)$ to $1.96\times\hat{\sigma}_{f_{1}}\left( m,L \right)$. Therefore, the CI-based detection criterion also means detection when the absolute value of the pseudo-observed S/N ratio, $\frac{G_{f_{1}o}\left( t_{0},m,L \right)}{\hat{\sigma}_{f_{1}}\left( m,L \right)}$, for a given month m, starting year t_o_, and time window of L years, was 1.96 or greater.

## Attribution

To attribute a detected pseudo-observed signal to a set of external forcing agents (f_2_), we first constructed the 95% CI of the distribution of the f_2_ forced signals using the Gaussian assumption. For any given set of forcing agents f_2_, month m, starting year t_0_, and length of time window L, the 95% CI was $G_{f_{2}f_{1}}\left( t_{0},m,L \right)-1.96\times D_{f_{2}f_{1}}\left( t_{0},m,L \right)$ to $G_{f_{2}f_{1}}\left( t_{0},m,L \right)+1.96\times D_{f_{2}f_{1}}\left( t_{0},m,L \right)$, where $G_{f_{2}f_{1}}\left( t_{0},m,L \right)$ was the weighted ensemble average and $D_{f_{2}f_{1}}\left( t_{0},m,L \right)$ the standard deviation of the signals $G_{f_{2}f_{1}\mathrm{di}}\left( t_{0},m,L \right)$ over all the f_2_-forced models (d) and ensemble members (i). The calculation of the weighted averages was the same as for the fingerprint calculation (Section 2.2). The calculation of the standard deviations did not use any weighting, which was consistent with the calculation of the noise term (Section 2.3). If the detected pseudo-observed signal lay within the 95% CI of the distribution of the f_2_ forced signals, we deemed the detected signal attributable to the set of forcing agents f_2_.

Additionally, for each simulated signal, the corresponding simulated S/N ratio was defined as $\frac{G_{f_{2}f_{1}\mathrm{di}}\left( t_{0},m,L \right)}{\hat{\sigma}_{f_{1}}\left( m,L \right)}$. The 95% CI of the distribution of f_2_-forced simulated S/N ratios was constructed analogously to the 95% CI of the f_2_ signals, i.e. from $\frac{G_{f_{2}f_{1}}\left( t_{0},m,L \right)}{\hat{\sigma}_{f_{1}}\left( m,L \right)}-1.96\times\frac{D_{f_{2}f_{1}}\left( t_{0},m,L \right)}{\hat{\sigma}_{f_{1}}\left( m,L \right)}$ to $\frac{G_{f_{2}f_{1}}\left( t_{0},m,L \right)}{\hat{\sigma}_{f_{1}}\left( m,L \right)}+1.96\times\frac{D_{f_{2}f_{1}}\left( t_{0},m,L \right)}{\hat{\sigma}_{f_{1}}\left( m,L \right)}$. Those 95% CI and the ensemble average $\frac{G_{f_{2}f_{1}}\left( t_{0},m,L \right)}{\hat{\sigma}_{f_{1}}\left( m,L \right)}$ were used to represent the “raw” S/N ratios in main text Fig. 4. Note that main text Fig. 3 shows the original pseudo-observed signals and 95% CI of the simulated signals and unforced trends, not S/N ratios.

# Sensitivity analysis on the results of D&A

We conducted the main D&A analysis on the 3-month, Gaussian mixture distribution-fitted SSI of the pseudo-observation, Mean NonCMIP, and calculated the signals and noises using all the available ensemble members under each forcing (Table 1). This setup is reported in all the figures and tables except Supplementary Tables 4–7. For sensitivity analysis on the D&A results, we performed the following variations:

1. using alternative timescales (1 and 6 months) and distributional fits (Weibull distribution) to calculate the SSI,
2. using the SSI of individual merged soil moisture product (Mean ORS, OLC ORS, and EC ORS) to conduct the D&A,
3. using the average SSI of alternative merged soil moisture products (Mean Products, instead of Mean NonCMIP) to conduct the D&A, and
4. using only the CMIP6 models and ensemble members that overlapped between the piControl and the ALL, GHG, or AER simulations (Table 3) to conduct the D&A.

The results of these variations are reported in Supplementary Tables 4–7.

# Physical justification for the emergent constraint relationship between modeled historical and future S/N ratios

Emergent constraint relationship means that the historical simulated variable(s) of an ESM is related to the future simulated variable of interest of the same ESM, and a statistically significant regression relationship can be constructed across the ESMs by treating the historical-future pair of each ESM as a data point in the regression. Past studies already demonstrated emergent constraint relationships between the historical and future trends in the drivers of soil moisture (e.g., warming rates^23^, LAI, gross primary productivity^24^), and historical and future drying in soil moisture in the northern mid-latitudes^25^. We also found temporally relatively stable zonal patterns of correlations between the drivers of soil moisture and the SSI in the ALL simulations (Figure 12). Therefore, it is reasonable to believe that emergent constraint relationships existed between the historical and future trends in SSI in the ALL simulations. Furthermore, the S/N ratios, by definition, were closely related to the zonal trends in SSI (Section 2). Therefore, it is reasonable to conjecture that emergent constraint relationships existed between the historical and future S/N ratios of the ALL simulations on the ALL fingerprint. This conjecture can then be verified by testing the statistical significance of the emergent constraint regressions.

We also demonstrate the above conjecture using the example of warming rates. If the ESMs with higher (lower) historical warming rates continue to have higher (lower) future warming rates, and the ESMs with higher (lower) historical sensitivities of the S/N ratios to the warming rates continue to have higher (lower) future sensitivities, the ESMs with higher (lower) historical S/N ratios should continue to have higher (lower) future S/N ratios. Past study already demonstrated the historical-future relationship in warming rates^23^. Figure 9 shows the relationship between the historical and future sensitivities using the S/N ratios based on 3-month SSI over the 2025–2070 future period as an example. Although the linear relationships of December–April for the 0–100 cm soil layer did not have positive slopes, the constrained S/N ratios of these months were very close to, and therefore no worse than, the model average S/N ratios (main text Fig. 4). In May–August, when the constrained and model average future S/N ratios were considerably different (main text Fig. 4), the positive linear relationships are always clear. We found similar results to Figure 9 for the S/N ratios based on 3-month SSI for all the future periods that were between 2020–2100 and 46-year long.

# Relationship between the S/N ratio and the trends in the SSI under the ALL forcing

Eq. (3) in Section 2 can be rewritten in vector form. We use $\mathbf{S}_{f_{1}\mathrm{di}}\left( m \right)$ to denote the matrix containing the spatiotemporal series of zonally averaged SSI of ESM d, ensemble member i, under the forcing f_1_ = ALL, as follows:

|  | $\mathbf{S}_{\mathbf{f}_{\mathbf{1}}\mathbf{di}}\left( m \right)\mathbf{=}\left[ \begin{matrix} S_{f_{1}\mathrm{di}}\left( \theta_{1},m,t_{1} \right) & \boldsymbol{\cdots} & S_{f_{1}\mathrm{di}}\left( \theta_{K},m,t_{1} \right) \\ \boldsymbol{\vdots} & \boldsymbol{\ddots} & \boldsymbol{\vdots} \\ S_{f_{1}\mathrm{di}}\left( \theta_{1},m,t_{T} \right) & \boldsymbol{\cdots} & S_{f_{1}\mathrm{di}}\left( \theta_{K},m,t_{T} \right) \end{matrix} \right]$ | (4) |
| --- | --- | --- |

where $\theta_{1}$, $\theta_{2}$, …, $\theta_{K}$ (K = 25) denote the 5° latitudinal bands centered around 50°S, 45°S, …, 70°N, and the time steps $t_{1}$, $t_{2}$, …, $t_{T}$ (T = 130) denote the years 1971, 1972, …, 2100 in the ALL simulations. The month m is sometimes dropped in subsequent notations because the descriptions below can be applied in the same way to any month of the year. We use $\mathbf{S}_{f_{1}}$ to denote the weighted average of $\mathbf{S}_{f_{1}\mathrm{di}}\left( m \right)$ across the ESMs and ensemble members, and use $\mathbf{A}\mathbf{=}\mathrm{diag}\left( \left[ \begin{matrix} a\left( \theta_{1} \right) & \boldsymbol{\ldots} & a\left( \theta_{K} \right) \end{matrix} \right] \right)$ to denote the diagonal matrix of weights proportional to the land surface area of each latitudinal band. The eigenvalue decomposition of the covariance matrix of $\mathbf{S}_{f_{1}}$ can be written as Eq. (5).

|  | $\boldsymbol{\Sigma}_{f_{1}}\mathbf{=}\frac{1}{T-1}\left( \mathbf{S}_{f_{1}}\mathbf{A} \right)^{\mathbf{'}}\left( \mathbf{S}_{f_{1}}\mathbf{A} \right)\mathbf{=}\sum_{k=1}^{K} \lambda_{k}\mathbf{w}_{\mathbf{k}}\mathbf{w}_{\mathbf{k}}^{\mathbf{'}}\boldsymbol{=W\Lambda}\mathbf{W}^{\mathbf{'}}$ | (5) |
| --- | --- | --- |

where the apostrophe denotes the transposition of a matrix or column vector, $\lambda_{k}$ is the k^th^ eigenvalue, $\mathbf{w}_{\mathbf{k}}$ is the k^th^ eigenvector, $\mathbf{W}$ is the matrix whose k^th^ column is $\mathbf{w}_{\mathbf{k}}$, and $\boldsymbol{\Lambda}$ is the diagonal matrix whose k^th^ diagonal element is $\lambda_{k}$. The EOF fingerprint of the D&A analysis, $F_{f_{1}}\left( \theta,m \right)$, is identical to the leading eigenvector $\mathbf{w}_{\mathbf{1}}$, Eq. (6).

|  | $\mathbf{w}_{1}\mathbf{=}\left[ \begin{matrix} F_{f_{1}}\left( \theta_{1},m \right) & \boldsymbol{\ldots} & F_{f_{1}}\left( \theta_{K},m \right) \end{matrix} \right]^{\mathbf{'}}$ | (6) |
| --- | --- | --- |

Therefore, the vector form of Eq. (3) is Eq. (7).

|  | $\mathbf{z}_{1di}=\mathbf{S}_{f_{1}\mathrm{di}}\mathbf{A}\mathbf{w}_{1}=\left[ \begin{matrix} \sum_{k=1}^{K} S_{f_{1}\mathrm{di}}\left( \theta_{k},m,t_{1} \right)\cdot a\left( \theta_{k} \right)\cdot F_{f_{1}}\left( \theta_{k},m \right) \\ \ldots\\ \sum_{k=1}^{K} S_{f_{1}\mathrm{di}}\left( \theta_{k},m,t_{T} \right)\cdot a\left( \theta_{k} \right)\cdot F_{f_{1}}\left( \theta_{k},m \right) \end{matrix} \right]$ | (7) |
| --- | --- | --- |

where the f_2_ in Eq. (3) is replaced by f_1_ because we are only concerned with the projection of the ALL simulation on the ALL fingerprint here, and $\mathbf{z}_{\mathbf{1}\mathbf{di}}$ is the projection of the zonally averaged SSI on the leading eigenvector (the subscript 1 means projection on $\mathbf{w}_{\mathbf{1}}$), meaning $\mathbf{z}_{1di}\mathbf{=}\left[ \begin{matrix} Z_{f_{1}f_{1}\mathrm{di}}\left( m,t_{1} \right) & \boldsymbol{\ldots} & Z_{f_{1}f_{1}\mathrm{di}}\left( m,t_{T} \right) \end{matrix} \right]^{\mathbf{'}}$. Analogous to Eq. (7), we can write the projections of the zonally averaged SSI on the other eigenvectors as $\mathbf{z}_{\mathrm{kdi}}=\mathbf{S}_{f_{1}\mathrm{di}}\mathbf{A}\mathbf{w}_{k}$, $k=2,\ldots,25$. In matrix notation, the projection operations can be written in Eq. (8).

|  | $\mathbf{Z}_{\mathbf{di}}\mathbf{=}\mathbf{S}_{f_{1}\mathrm{di}}\mathbf{AW}$ | (8) |
| --- | --- | --- |

where $\mathbf{Z}_{\mathbf{di}}\mathbf{=}\left[ \begin{matrix} \mathbf{z}_{\mathbf{1}\mathrm{di}} & \boldsymbol{\ldots} & \mathbf{z}_{\mathrm{Kdi}} \end{matrix} \right]$. Because the eigenvectors obtained in the EOF analysis are mutually orthogonal unit vectors^22^, meaning $\mathbf{W}\mathbf{W}^{\mathbf{'}}\mathbf{=}\mathbf{W}^{\mathbf{'}}\mathbf{W=I}$, where $\mathbf{I}$ is the identity matrix, post-multiplying both sides of Eq. (8) by $\mathbf{W}^{\mathbf{'}}$, and removing the area-based weighting by post-multiplying with $\mathbf{A}^{\mathbf{-1}}=\mathrm{diag}\left( \left[ \begin{matrix} 1/{a\left( \theta_{1} \right)} & \boldsymbol{\ldots} & 1/{a\left( \theta_{K} \right)} \end{matrix} \right] \right)$ result in Eq. (9).

|  | $\mathbf{Z}_{\mathbf{di}}\mathbf{W}^{\mathbf{'}}\mathbf{A}^{\mathbf{-1}}\mathbf{=}\sum_{k=1}^{K} \left[ \mathbf{z}_{\mathrm{kdi}}\left( \mathbf{w}_{k}^{'}\mathbf{A}^{\mathbf{-1}} \right) \right]\mathbf{=}\mathbf{S}_{f_{1}\mathrm{di}}\mathbf{AW}\mathbf{W}^{\mathbf{'}}\mathbf{A}^{\mathbf{-1}}\mathbf{=}\mathbf{S}_{f_{1}\mathrm{di}}$ | (9) |
| --- | --- | --- |

Eq. (9) shows that the matrix containing the spatiotemporal series of zonally averaged SSI for any ESM d and ensemble member i (right-most term) can be viewed as the sum of the dot products between the projections of the zonally averaged SSI on the eigenvectors and the eigenvectors themselves, scaled by the diagonal matrix $\mathbf{A}^{\mathbf{-1}}$ (second term to the left). Finally, the least-squares formula for estimating the slopes of linear regression is a linear transformation^26^—that is, let $\mathbf{X}$ be the matrix whose first column is 1’s, and second column is time, then estimating the linear least squares trend in each column of $\mathbf{S}_{f_{1}\mathrm{di}}$ simply involves pre-multiplying by the matrix $\mathbf{H=}\left( \mathbf{X}^{\mathbf{'}}\mathbf{X} \right)^{\mathbf{-1}}\mathbf{X}^{\mathbf{'}}$. Pre-multiplying this matrix on both sides of Eq. (9), and separating out the term involving projection on the leading eigenvector results in Eq. (10).

|  | $\left( \mathbf{H}\mathbf{z}_{1di} \right)\left( \mathbf{w}_{1}^{'}\mathbf{A}^{\mathbf{-1}} \right)+\sum_{k=2}^{K} \left[ \left( \mathbf{H}\mathbf{z}_{\mathrm{kdi}} \right)\left( \mathbf{w}_{k}^{'}\mathbf{A}^{\mathbf{-1}} \right) \right]\mathbf{=}\mathbf{H}\mathbf{S}_{f_{1}\mathrm{di}}$ | (10) |
| --- | --- | --- |

Although Eq. (10) is written for the trend over the entire time period t = 1, …, T, the vectors $\mathbf{z}_{1di}$, …, $\mathbf{z}_{\mathrm{kdi}}$ and the matrix $\mathbf{S}_{f_{1}\mathrm{di}}$ can be replaced by a subset of the time period without affecting the equality. Finally, $\mathbf{H}\mathbf{z}_{1di}$ is the trend in the projection vector $\mathbf{z}_{1di}$, and differs from the S/N ratio $\frac{G_{f_{1}f_{1}\mathrm{di}}\left( t_{0},m,L \right)}{\hat{\sigma}_{f_{1}}\left( m,L \right)}$ (Section 2.5) only by a constant scaling factor $\hat{\sigma}_{f_{1}}\left( m,L \right)$. Therefore, Eq. (10) can be further written as Eq. (11).

|  | $\frac{G_{f_{1}f_{1}\mathrm{di}}\left( t_{0},m,L \right)}{\hat{\sigma}_{f_{1}}\left( m,L \right)}\cdot\hat{\sigma}_{f_{1}}\left( m,L \right)\cdot\left( \mathbf{w}_{1}^{'}\mathbf{A}^{\mathbf{-1}} \right)+\mathbf{R}_{\mathrm{di}}\mathbf{=}\mathbf{H}\mathbf{S}_{f_{1}\mathrm{di}}$ | (11) |
| --- | --- | --- |

Eq. (11) shows that the month-by-latitude trends pattern in the SSI of any ESM (d) and ensemble member (i), under the ALL forcing, can be written as the sum of one S/N ratio-related component, which is proportional to the S/N ratio of this ESM and ensemble member, and a remainder component $\mathbf{R}_{\mathbf{di}}$, which is related to the trends in the projections on the non-leading eigenvectors ($\mathbf{R}_{\mathrm{di}}\mathbf{=}\sum_{k=2}^{K} \left[ \left( \mathbf{H}\mathbf{z}_{\mathrm{kdi}} \right)\left( \mathbf{w}_{k}^{'}\mathbf{A}^{\mathbf{-1}} \right) \right])$. Naturally, both sides of Eq. (11) can be averaged across the ensemble members of each ESM, and then across the ESMs, to relate the weighted average trend patterns to the weighted average S/N ratios.

Using the emergent constraint results, it is natural to replace the S/N ratio in the first term in Eq. (11) with the constrained S/N ratio as follows:

|  | $\frac{\mathcal{G}_{f_{1}f_{1}}\left( t_{0},m,L \right)}{\hat{\sigma}_{f_{1}}\left( m,L \right)}\cdot\hat{\sigma}_{f_{1}}\left( m,L \right)\cdot\left( \mathbf{w}_{1}^{'}\mathbf{A}^{\mathbf{-1}} \right)+\mathbf{R}_{\mathrm{di}}\mathbf{=}\mathbf{H}\mathcal{S}_{f_{1}\mathrm{di}}$ | (12) |
| --- | --- | --- |

where $\frac{\mathcal{G}_{f_{1}f_{1}}\left( t_{0},m,L \right)}{\hat{\sigma}_{f_{1}}\left( m,L \right)}$ is the S/N ratio after emergent constraint, and $\mathbf{H}\mathcal{S}_{f_{1}\mathrm{di}}$ is the adjusted trends in the SSI using the constrained S/N ratio. Because the physical interpretation of the S/N ratio is the response of the SSI to the ALL forcing, this replacement can be interpreted as correcting the forced response-related component of bias in the projected SSI trends. This bias-correction does not consider biases in the fingerprint $\mathbf{w}_{1}^{'}$, because there is no observational equivalent to $\mathbf{w}_{1}$ in the D&A framework used in this study (Section 2). Possible correlations between the biases in the S/N ratio and the biases in the two quantities related to natural variability ($\hat{\sigma}_{f_{1}}\left( m,L \right)$ and $\mathbf{R}_{\mathrm{di}}$) were assumed to be small based on the rationale that the effect of natural variability has been removed via dividing the signal $G_{f_{1}f_{1}\mathrm{di}}\left( t_{0},m,L \right)$ by $\hat{\sigma}_{f_{1}}\left( m,L \right).$

The results of repeatedly applying Eqs. (11) and (12) on the zonally averaged 3-month SSI on all the months of the year and multiple future periods are shown in Figure 10 and Figure 11. The rows in the figures correspond to terms in Eqs. (11) and (12) as follows:

- Row 1 (panels a–e): $\mathbf{H}\mathbf{S}_{f_{1}\mathrm{di}}$: the original trends in SSI before emergent constraint
- Row 2 (panels f–j): $\frac{G_{f_{1}f_{1}\mathrm{di}}\left( t_{0},m,L \right)}{\hat{\sigma}_{f_{1}}\left( m,L \right)}\cdot\hat{\sigma}_{f_{1}}\left( m,L \right)\cdot\left( \mathbf{w}_{1}^{'}\mathbf{A}^{\mathbf{-1}} \right)$: the original S/N ratio-related component of the trends
- Row 3 (panels k–o): $\mathbf{R}_{\mathrm{di}}$: the original remainder component of the trends
- Row 4 (panels p–t): $\frac{\mathcal{G}_{f_{1}f_{1}}\left( t_{0},m,L \right)}{\hat{\sigma}_{f_{1}}\left( m,L \right)}\cdot\hat{\sigma}_{f_{1}}\left( m,L \right)\cdot\left( \mathbf{w}_{1}^{'}\mathbf{A}^{\mathbf{-1}} \right)$: the constrained S/N ratio-related component of the trends based on the constrained S/N ratios
- Row 5 (panels u–y): $\mathbf{H}\mathcal{S}_{f_{1}\mathrm{di}}$: the adjusted trends in SSI based on the constrained S/N ratios and the original remainder component.

The coloring in rows 1–3 reflect the weighted average of the terms across the ESMs and ensemble members, and the hatching reflects the variability across the ESMs and ensemble members. Because the S/N ratios are scalars and do not vary with latitude, the hatching in the row 2 of Figure 10 and Figure 11 is the same for all the latitudes in the same month. The coloring in row 4 reflects the values of the term calculated from the point estimates of the constrained S/N ratios, and the hatching is based on the 90% and 80% CIs of term, calculated from the 90% and 80% CIs of the constrained S/N ratios. The coloring in row 5 reflects the weighted average of the term across the ESMs and ensemble members, but the hatching only reflects the variability in the $\mathbf{R}_{\mathrm{di}}$ component across the ESMs and ensemble members, not the uncertainty in the constrained S/N ratios.

# Supplementary Figures


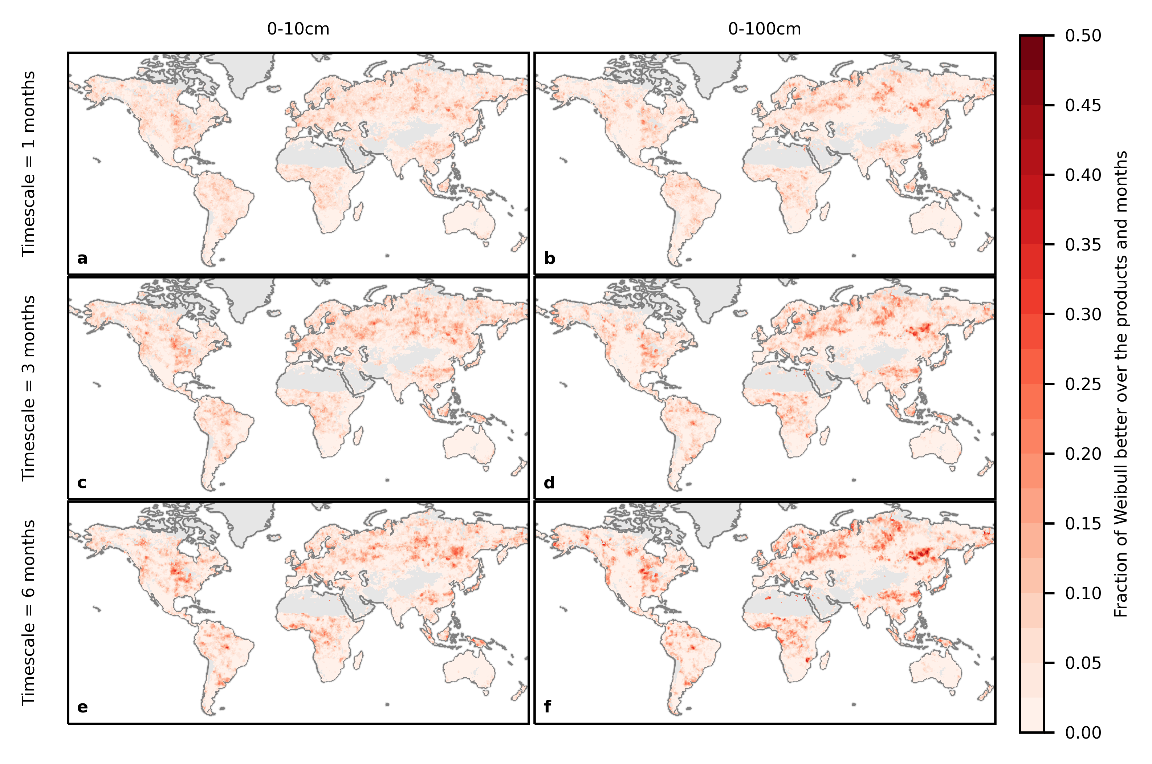


**Figure 1. The proportion of products (Mean ORS, OLC ORS, and EC ORS) and months of the year (January–December) where the log-likelihood of fitting a Weibull distribution is higher than fitting a Gaussian mixture distribution.**


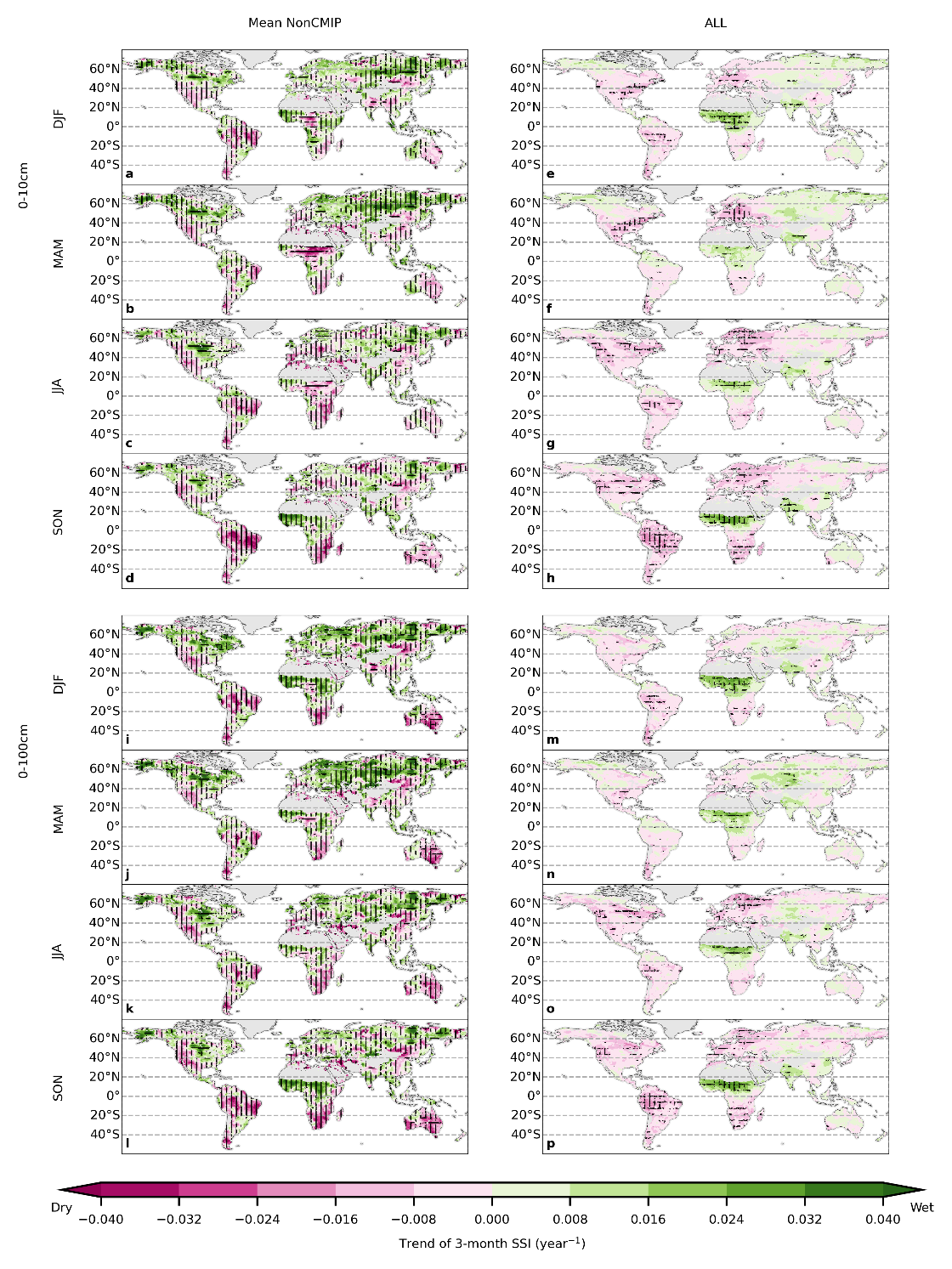


**Figure 2. The 1971–2016 trends in the 0–10 cm and 0–100 cm 3-month SSI of the pseudo-observation (Mean NonCMIP) and the ALL simulations.** The trends of the ALL simulations were first calculated on the individual ensemble members before being averaged for display in panels e–h and m–p. In panels a–d and i–l, the vertically hatched area indicates that the trends of the pseudo-observation had the same signs as the averages of the ALL simulations; the horizontally hatched area indicates that the former had the opposite signs to the latter and were outside the 95% CIs of the trends of the ALL simulations. In panels e–h and m–p, the vertical hatching indicates at least 90% of the simulations agreed on the signs of the trends, and the horizontal hatching 80%.


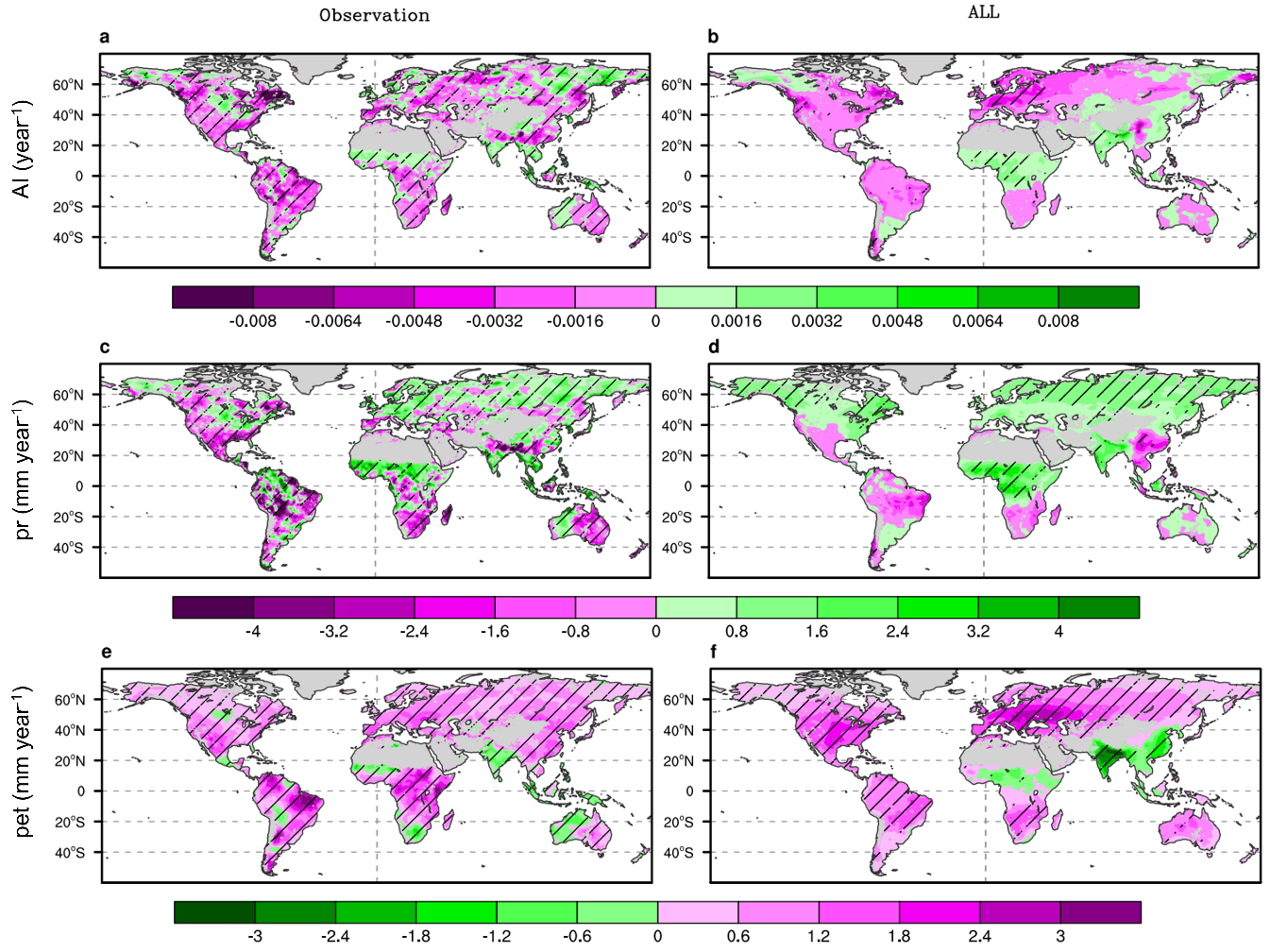


**Figure 3. The 1971–2014 trends in the annual mean aridity index (AI, defined as the ratio of precipitation to potential evapotranspiration), annual total precipitation (pr), and annual total potential evapotranspiration (pet) of observations and the ALL simulations.** The observed precipitation data sets were from the Climate Research Unit v4.05^27^, the Climate Prediction Center National Centers of the Environmental Prediction^28^, the University of Delaware^29^, and the Global Precipitation Climatology Centre^30^. The observed potential evapotranspiration was calculated using a modified Penmann-Monteith model that considered the impacts from changing CO_2_^31^ and data from the NOAA-CIRES Twentieth Century Reanalysis^32^, the ECMWF Reanalysis v5^33^, and the Global Land Data Assimilation System^34^. The displayed observation trends were the averaged trends over the twelve combinations of precipitation and reanalysis data sets (panel a), the four precipitation data sets (panel c), and the three reanalysis data sets (panel e). The displayed ALL trends were the averaged trends over the ALL simulations. Hatching indicates at least 75% agreement on the signs of the trends.


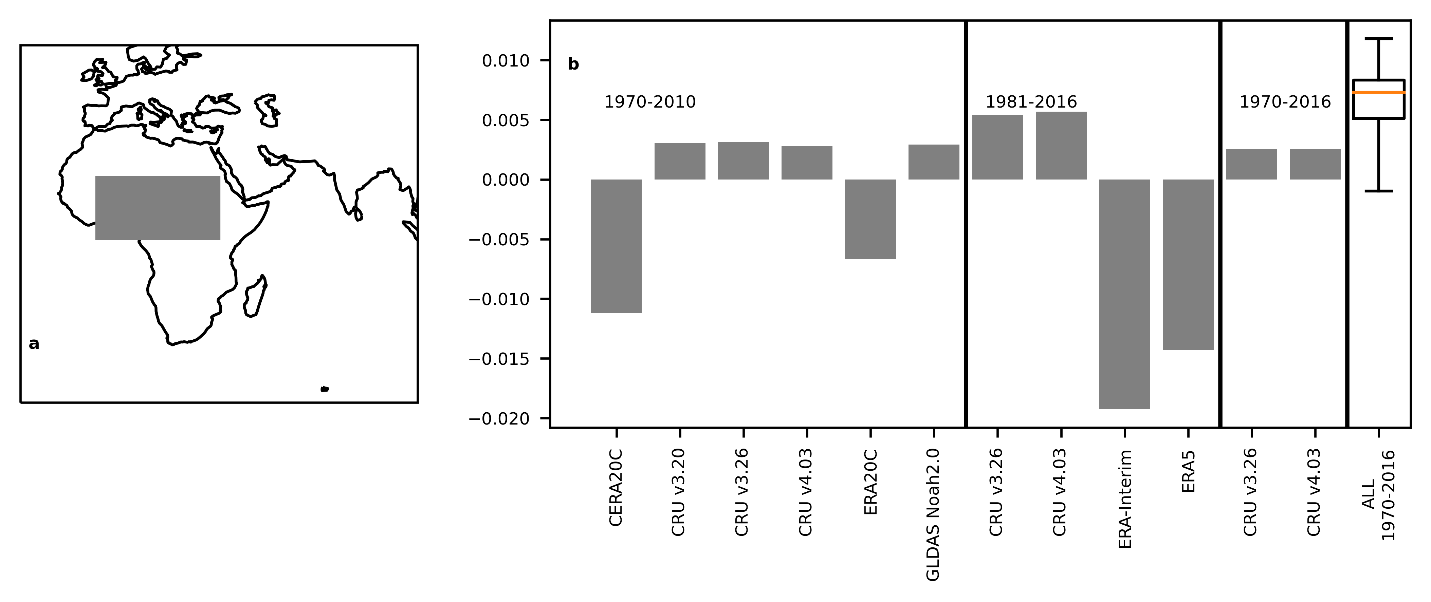


**Figure 4. Historical trends in the annual mean precipitation (mm day^-1^ year^-1^) over the Sahel region. a** Extent of the region (0°N–20°N, 5°W–40°E). **b** The trends in the individual drivers of the source soil moisture data sets used to create the merged soil moisture data sets^1^ and the CMIP6 ALL simulations. The drivers of the source soil moisture data sets include several versions of the Climate Research Unit gridded rain gauge observations (CRU v3.20, CRU v3.26, CRU v4.03) and several reanalysis (CERA20C, ERA20C, GLDAS Noah 2.0, ERA-Interim, ERA5)^1^. The source soil moisture data sets do not all cover the 1971**–**2016 period^1^. Hence the comparison of trends shows a few different time periods.


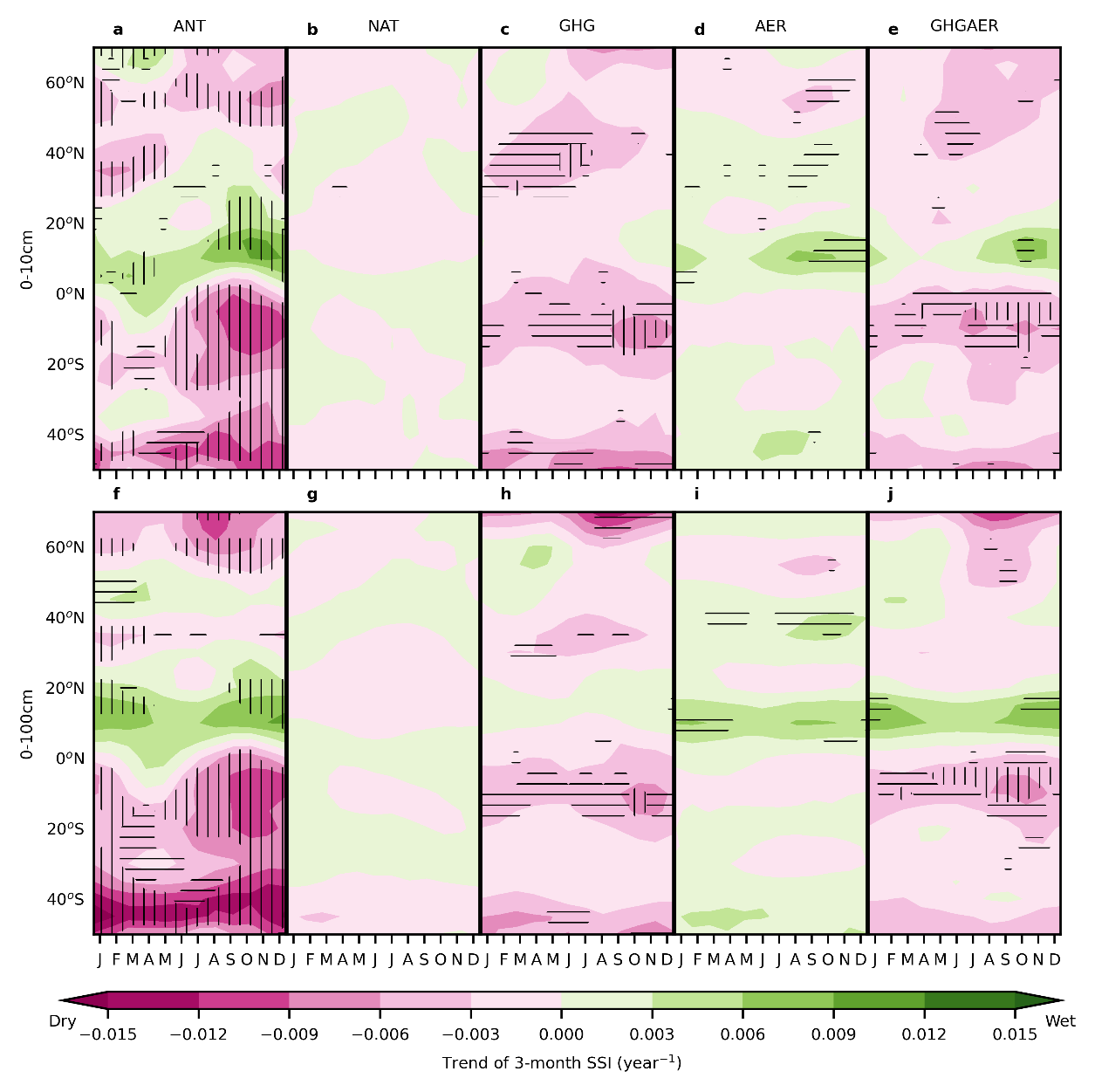


**Figure 5.** **Latitude-by-month 1971–2016 trends in the zonally averaged 3-month SSI of the historical ANT, NAT, GHG, AER, and GHGAER simulations.** The trends were calculated on individual model ensemble members and then averaged for display. Vertical hatching indicates at least 90% of the simulations agreed on the signs of the trends, and horizontal hatching 80%.


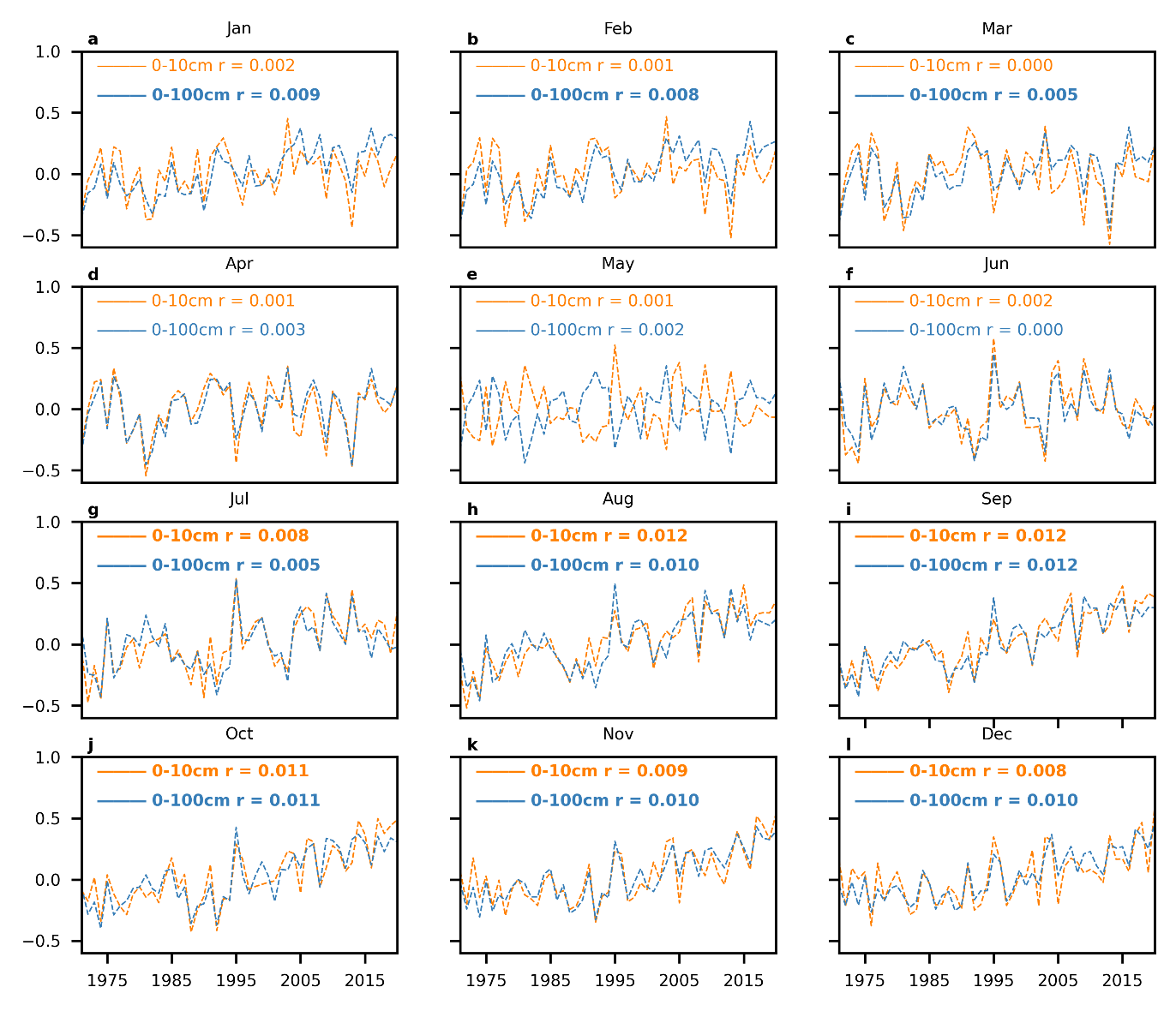


**Figure 6. The principal components associated with the AER fingerprints of each month and soil layer.** The texts after each legend entry denotes the trend in the principal component over 1971–2016. Bold text indicate significance at the 95% confidence level.


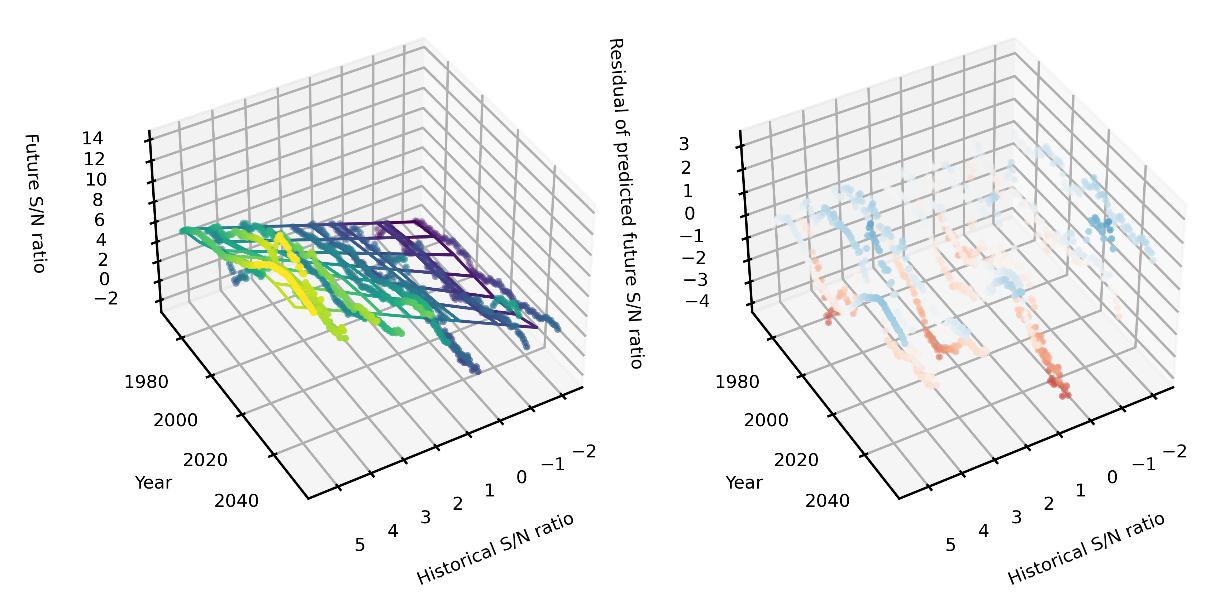


**Figure 7. Emergent relationships between the historical and future S/N ratios of the ALL simulations on the ALL fingerprints for the 0–10 cm soil layer, May.** The “year” axis denotes the starting year of the period of the future S/N ratios, which always spanned 46-year time windows. The dots indicate the S/N ratios, and the meshgrid indicates the fitted GAM model.


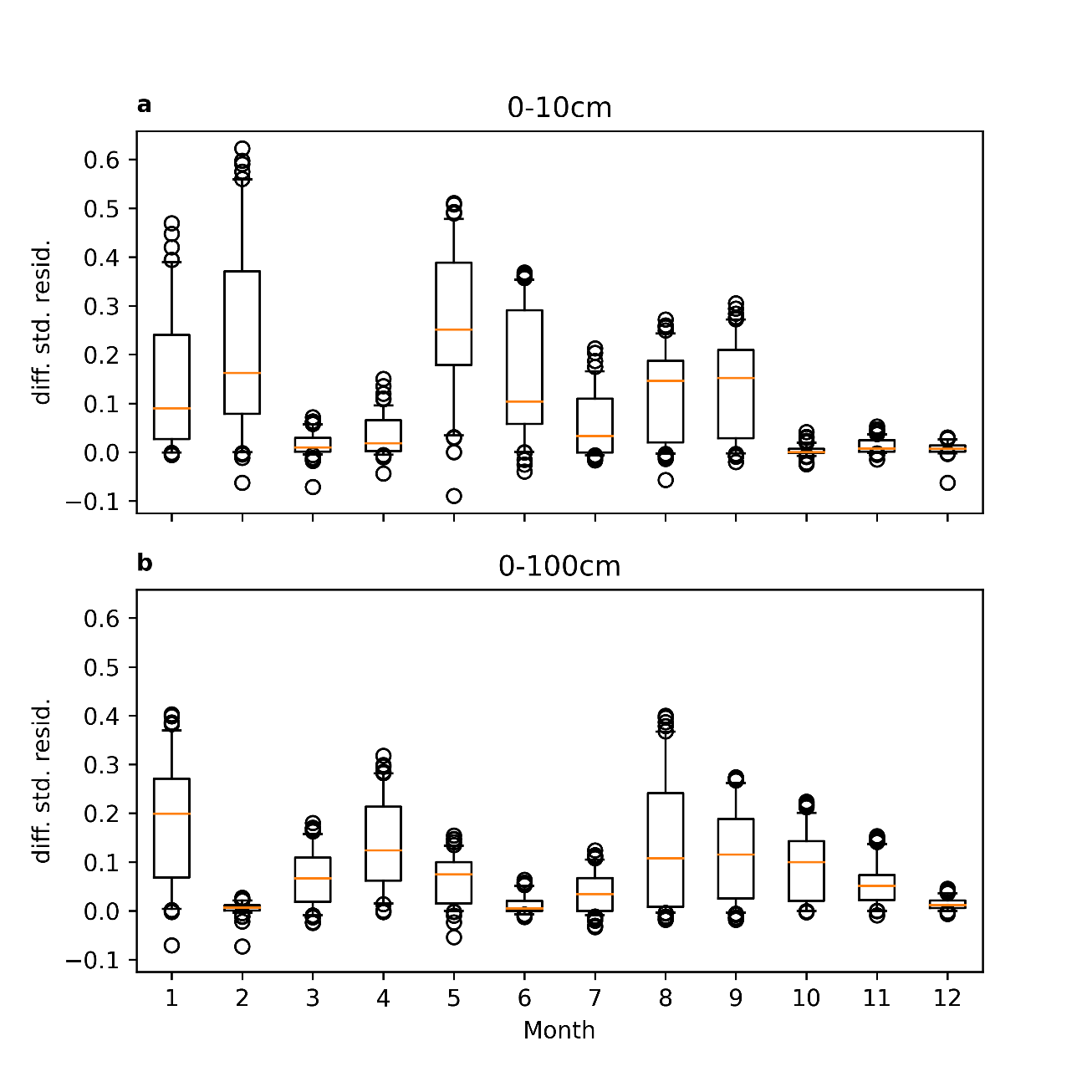


**Figure 8. Differences in the standard deviations of the residuals of fit (diff. std. resid.) between applying a separate linear regression between the historical ALL-forced S/N ratios and the ALL-forced S/N ratios of each future period, and applying one generalized additive model over all the future periods (the former minus the latter)**. The box plots indicate the median (middle line), 25^th^ and 75^th^ percentiles (box), 5^th^ and 95^th^ percentiles (whiskers), as well as outliers (single points) of the differences of all the future periods (1972–2017, 1973–2018, …, 2055–2100). All the S/N ratios were projected on the ALL fingerprints.


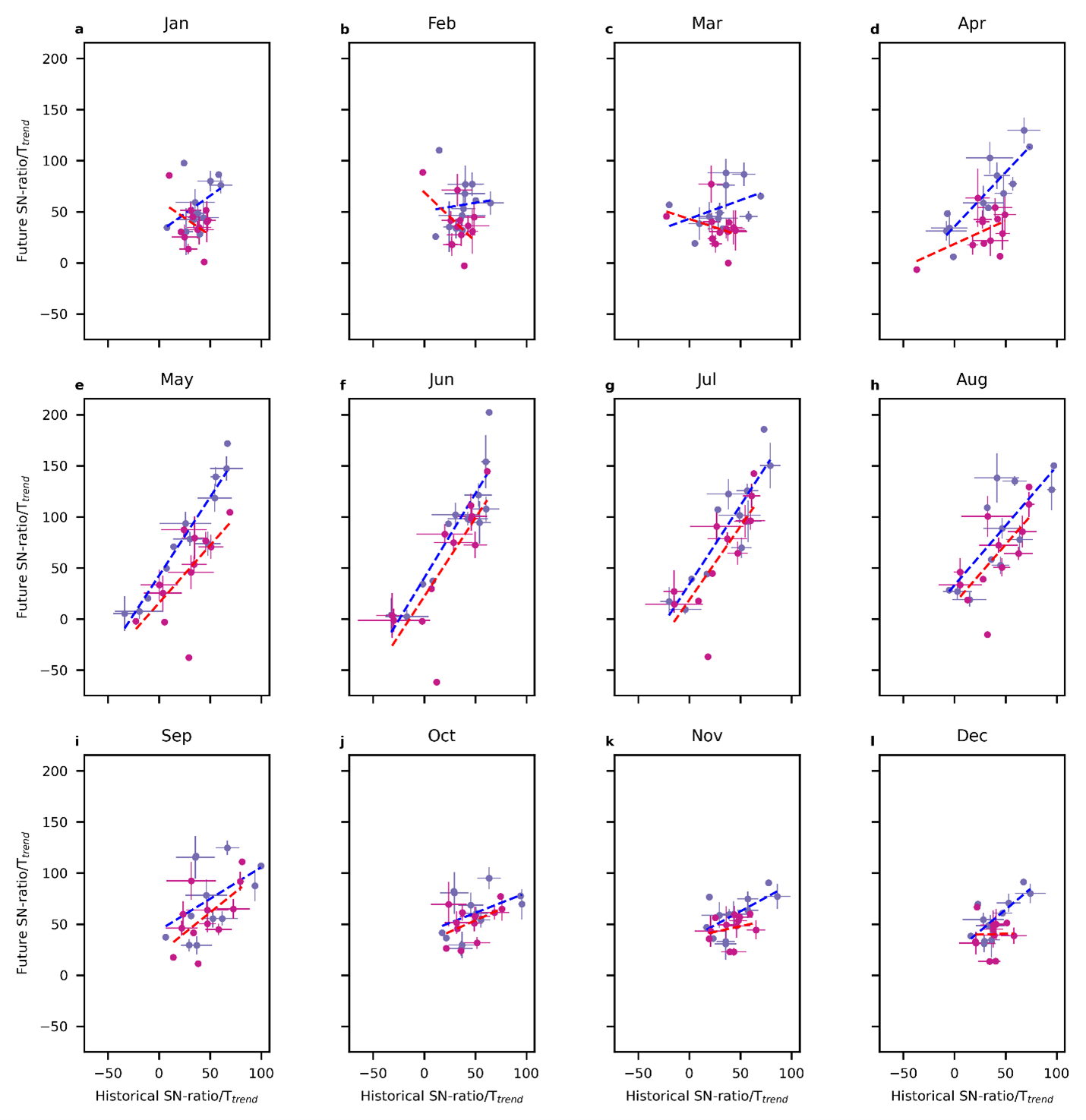


**Figure 9. Relationships between the historical (1971**–**2016) and the future (2025–2070) sensitivities of the ALL-forced S/N ratios to modeled warming rates (T_trend_, ^o^C year^-1^), for each soil layer and month of the year.** Blue (red) dots and fitted lines represent the 0–10 cm (0–100 cm) soil layer. Each data point corresponds to one model, with the dot indicating the mean and the error bars ±1 standard deviation across the model’s ensemble members. The warming rates were calculated by least squares on global mean temperature. All the S/N ratios were projected on the ALL fingerprints.


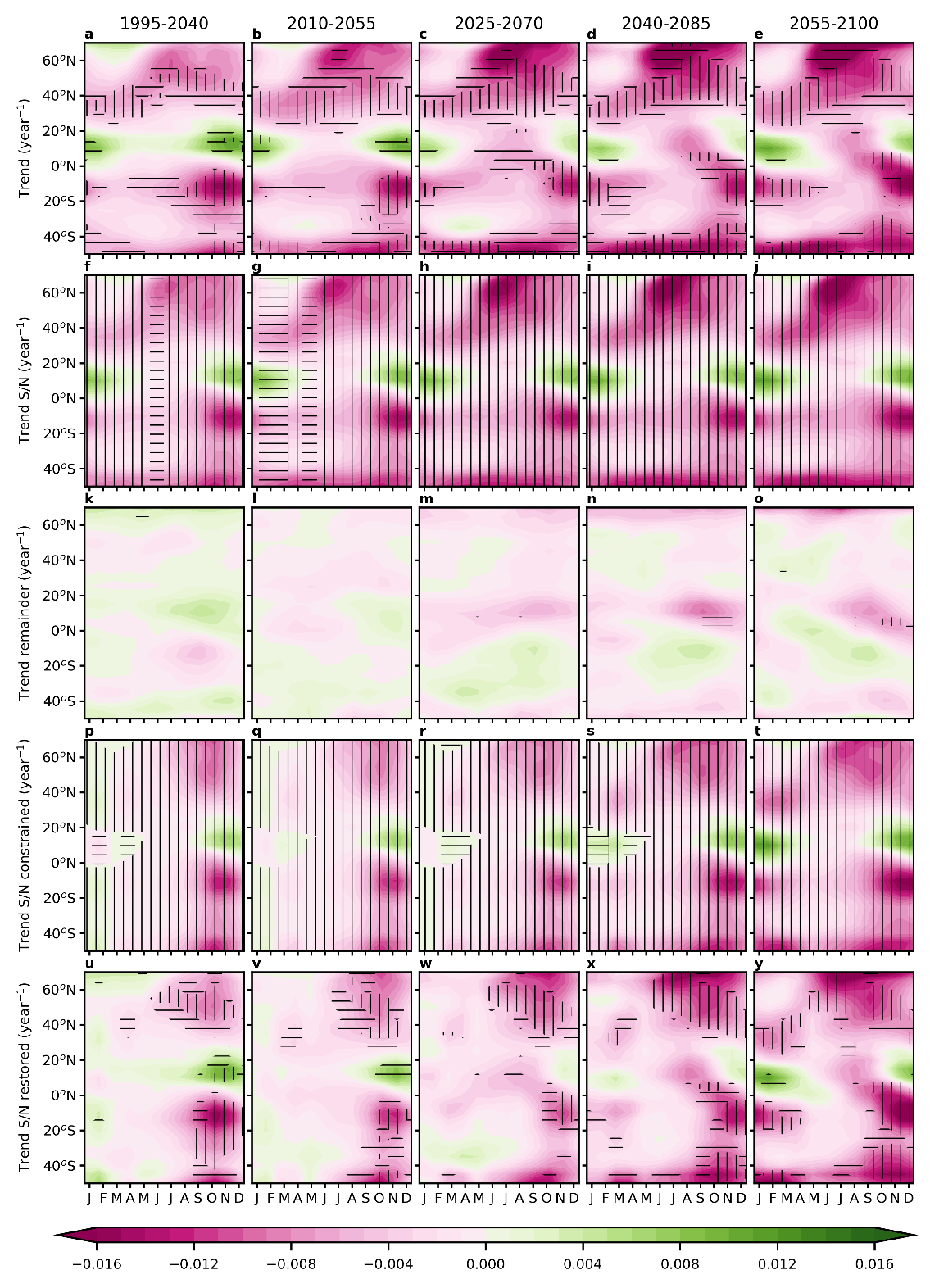


**Figure 10. Adjustment of the future 0–10 cm 3-month SSI trends based on the constrained ALL-forced S/N ratios: a–e** The latitude-by-month trends of the ALL simulations over multiple future time periods. **f–j** The original S/N ratio-related component of the trends. **k–o** The original remainder component of the trends. **p–t** The constrained S/N ratio-related component of the trends based on the constrained S/N ratios. **u–y** The adjusted trends based on the constrained S/N ratios and the original remainder component. The values shown in the panels a–o and u–y were averages across the ALL simulations. In the panels a–o and u–y, vertical hatching indicates at least 90% of the simulations agreed on the signs of the trends, and horizontal hatching 80%. The vertical hatching in the panels f–j and p–t indicates significant difference from zero at 90% confidence level, based on the CIs of the constrained S/N ratios, and the horizontal hatching 80%. All the S/N ratios were on the ALL fingerprints.


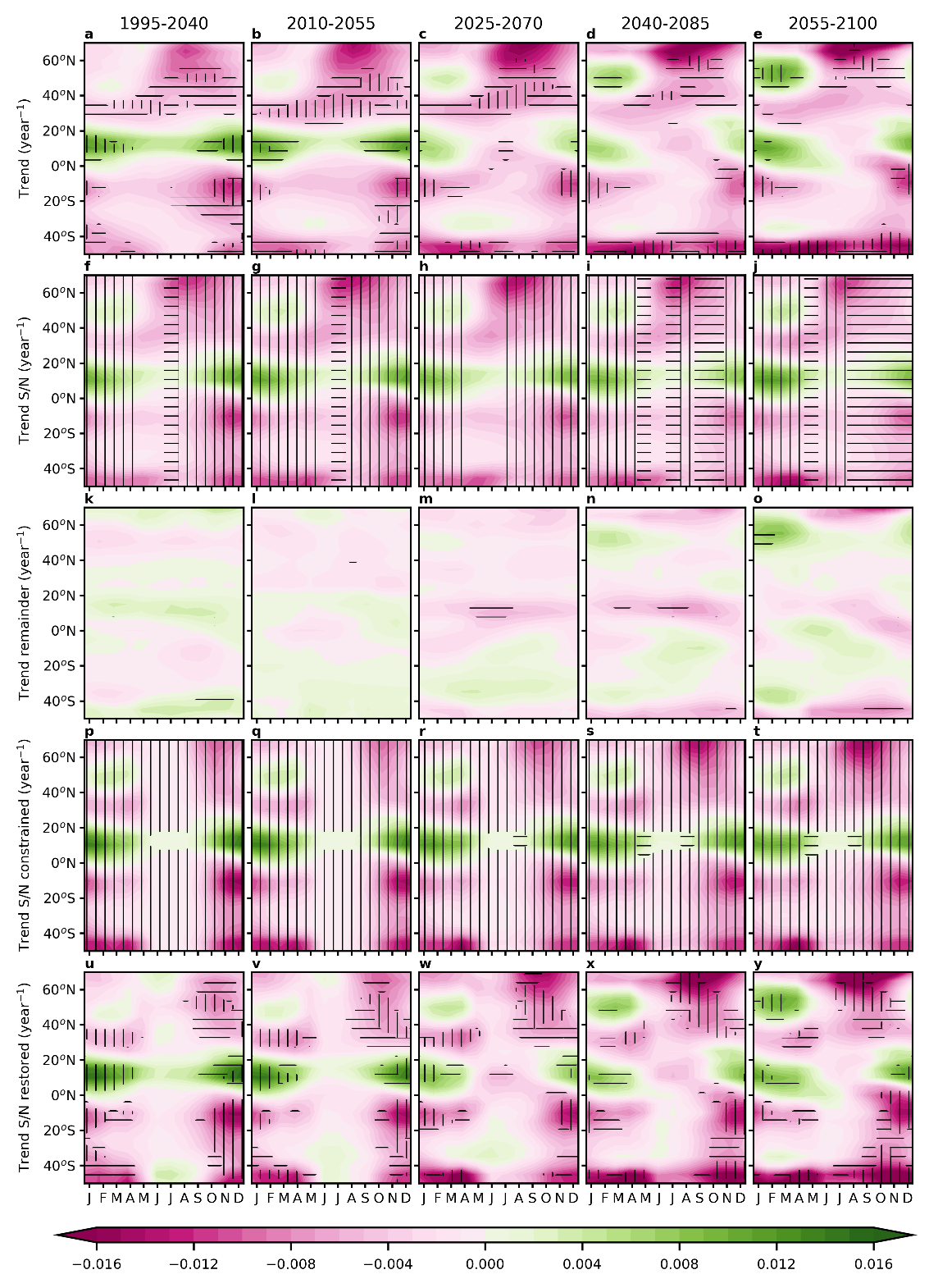


**Figure 11. The same as Figure 10 except for the 0–100 cm 3-month SSI.**


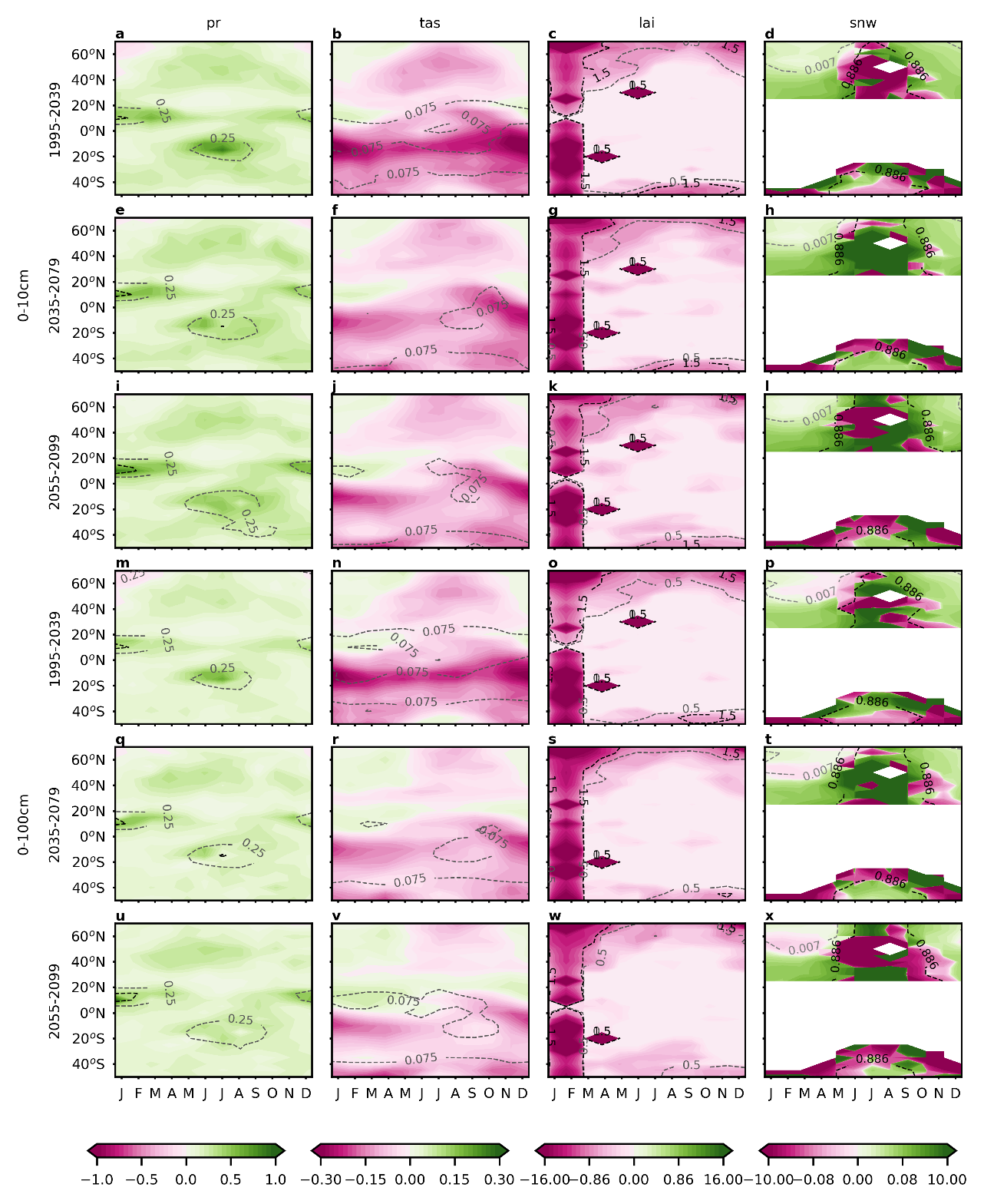


**Figure 12. Ridge regression**^35^ **coefficients between the 3-month SSI of each soil layer (0–10 cm, 0–100 cm) and the drivers of the SSI over three time periods.** The regression coefficient on lai was constrained to be positive-only. The regression coefficients on snw were masked in the latitude-months where the annual mean snw was less than 0.01 mm. The penalty parameters in the ridge regressions were chosen to be 0.1 based on goodness-of-fit. The regressions were conducted individually for each latitude, month, and ALL simulation, and were averaged over the ensemble members for display. The dotted contours indicate the standard deviations in the regression coefficients across the ALL ensemble members. pr – precipitation, tas – temperature, lai ­– leaf area index, snw – snow water equivalent.


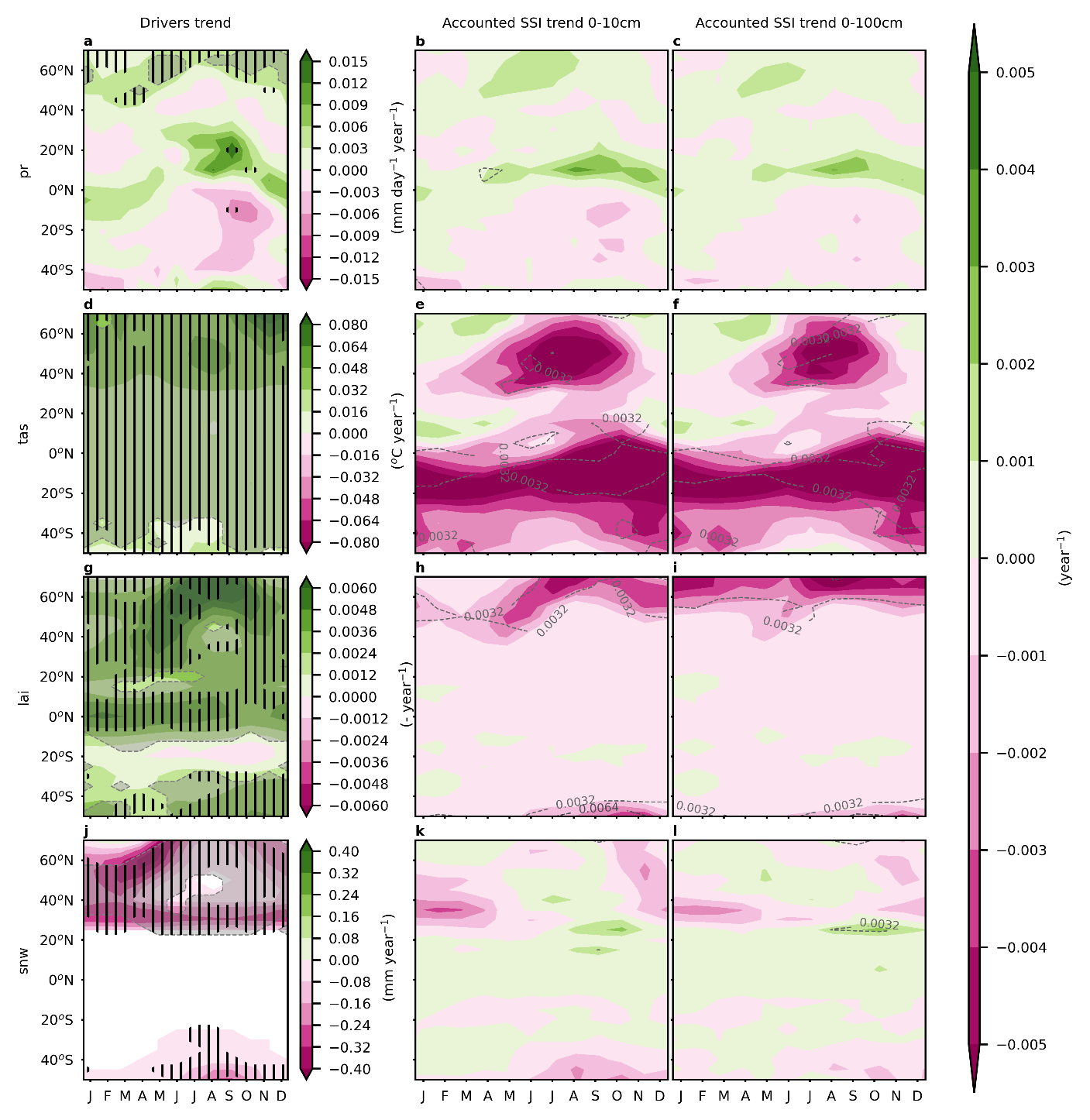


**Figure 13. Trends in the drivers of 3-month SSI and the accounted-for trends in the 0–10 cm and 0–100 cm SSI by these drivers during 1971–2016. a, d, g, j** Coloring represents the average trends over the ALL simulations. Vertical hatching indicates more than 90% of the simulations agreed on the signs of the trend. Gray shading indicates more than 50% of the simulated trends were significantly different from zero at 95% confidence level. The trends in snw were masked in the latitude-months where the annual mean snw was less than 0.01 mm. **b, c, e, f, h, I, k, l** Coloring and dotted contours represents, respectively, the average and standard deviations of the accounted-for trends over the ALL simulations. The accounted-for trends were calculated as the ridge regression^35^ coefficients (Figure 12) multiplied by the trends in the drivers. pr – precipitation, tas – surface air temperature, lai – leaf area index, snw – snow water equivalent.

# Supplementary Tables

Table 1. Summary of the seven merged soil moisture products. The abbreviations were constructed from initial letters.

| Name | Method | Observational constraint | Source data of soil moisture |
| --- | --- | --- | --- |
| Mean-ORS | Simple unweighted averaging | None | Offline land surface models^36,37^, reanalysis^33,34,38–44^, satellite^45^ (ORS) |
| OLC-ORS | Optimal linear combination^46,47^ (OLC) | In situ soil moisture measurements^48^ | ORS |
| EC-ORS | Emergent constraint^49^ (EC) | Observed gridded temperature and precipitation^50^ | ORS |
| EC-CMIP5 |  |  | CMIP5 ESMs^51^ |
| EC-CMIP6 |  |  | CMIP6 ESMs^5^ |
| EC-CMIP5+6 |  |  | CMIP5 and CMIP6 ESMs^5,51^ |
| EC-ALL |  |  | ORS, CMIP5 and CMIP6 ESMs^5,51^ (ALL) |

Table 2. Correspondence between the external forcings and the CMIP6 simulations used to represent the forcings. ALL—all anthropogenic and natural forcings, ANT—anthropogenic forcings only, NAT—natural forcings only, GHG—greenhouse gas only, AER—anthropogenic aerosols only, and GHGAER—both greenhouse gas and anthropogenic aerosols.

| External forcing | Description of the forcing agents^4^ | CMIP6 simulations | Number of models (ensemble members) | Years in simulation |
| --- | --- | --- | --- | --- |
| ALL | Well-mixed greenhouse gases, anthropogenic aerosols (BC, OC, SO_2_, SO_4_, NO_x_, NH_3_, CO, NMVOC), nitrogen deposition, ozone, stratospheric aerosols (volcano activities), solar irradiance, land use | Concatenated historical and SSP5-8.5 | 12 (84) | 1970–2100 |
| ANT | Well-mixed greenhouse gases, anthropogenic aerosols, nitrogen deposition, ozone, land use | ALL – hist-nat | 3 (12) | 1970–2020 |
| NAT | Solar irradiance, stratospheric aerosols (volcano activities) | hist-nat | 4 (27) | 1970–2020 |
| GHG | Well-mixed greenhouse gases | hist-GHG | 5 (30) | 1970–2020 |
| AER | Anthropogenic aerosols (BC, OC, SO_2_, SO_4_, NO_x_, NH_3_, CO, NMVOC) | hist-aer | 5 (28) | 1970–2020 |
| GHGAER | Well-mixed greenhouse gases, anthropogenic aerosols (BC, OC, SO_2_, SO_4_, NO_x_, NH_3_, CO, NMVOC) | hist-GHG + hist-aer | 5 (28) | 1970–2020 |
| Natural internal variability | No time evolving forcings | piControl | 17 (19) | Varies with ESM, last 200 years |

Table 3. List of the CMIP6 ESMs and their ensemble members used in the D&A.

| Model | Ensemble members in each experiment | | | | | |
| --- | --- | --- | --- | --- | --- | --- |
|  | historical + SSP5-8.5 | hist-GHG | hist-aer | hist-nat | piControl | historical |
| BCC-CSM2-MR | r1i1p1f1 | r1i1p1f1, r2i1p1f1, r3i1p1f1 | r1i1p1f1, r2i1p1f1, r3i1p1f1 | r1i1p1f1, r2i1p1f1, r3i1p1f1 | r1i1p1f1 | r1i1p1f1, r2i1p1f1, r3i1p1f1 |
| BCC-ESM1 | — | — | — | — | r1i1p1f1 | r1i1p1f1, r2i1p1f1, r3i1p1f1 |
| CanESM5 | r1i1p1f1, r1i1p2f1, r2i1p1f1, r2i1p2f1, r3i1p1f1, r3i1p2f1, r4i1p1f1, r4i1p2f1, r5i1p1f1, r5i1p2f1, r6i1p1f1, r6i1p2f1, r7i1p1f1, r7i1p2f1, r8i1p1f1, r8i1p2f1, r9i1p1f1, r9i1p2f1, r10i1p1f1, r10i1p2f1, r11i1p1f1, r11i1p2f1, r12i1p1f1, r12i1p2f1, r13i1p1f1, r13i1p2f1, r14i1p1f1, r14i1p2f1, r15i1p1f1, r15i1p2f1, r16i1p1f1, r16i1p2f1, r17i1p1f1, r17i1p2f1, r18i1p1f1, r18i1p2f1, r19i1p1f1, r19i1p2f1, r20i1p1f1, r20i1p2f1, r21i1p1f1, r21i1p2f1, r22i1p1f1, r22i1p2f1, r23i1p1f1, r23i1p2f1, r24i1p1f1, r24i1p2f1, r25i1p1f1, r25i1p2f1 | r1i1p1f1, r2i1p1f1, r3i1p1f1, r4i1p1f1, r5i1p1f1, r6i1p1f1, r7i1p1f1, r8i1p1f1, r9i1p1f1, r10i1p1f1 | r1i1p1f1, r2i1p1f1, r3i1p1f1, r4i1p1f1, r5i1p1f1, r6i1p1f1, r7i1p1f1, r8i1p1f1, r9i1p1f1, r10i1p1f1 | r1i1p1f1, r2i1p1f1, r3i1p1f1, r4i1p1f1, r5i1p1f1, r6i1p1f1, r7i1p1f1, r8i1p1f1, r9i1p1f1, r10i1p1f1 | r1i1p1f1, r1i1p2f1 | r1i1p1f1, r1i1p2f1, r20i1p1f1, r20i1p2f1, r21i1p1f1, r21i1p2f1, r22i1p1f1, r22i1p2f1, r23i1p1f1, r23i1p2f1, r24i1p1f1, r24i1p2f1, r25i1p1f1, r25i1p2f1, r2i1p1f1, r2i1p2f1, r3i1p1f1, r3i1p2f1, r4i1p1f1, r4i1p2f1, r5i1p1f1, r5i1p2f1, r6i1p1f1, r6i1p2f1, r7i1p1f1, r7i1p2f1, r8i1p1f1, r8i1p2f1, r9i1p1f1, r9i1p2f1, r10i1p1f1, r10i1p2f1, r11i1p1f1, r11i1p2f1, r12i1p1f1, r12i1p2f1, r13i1p1f1, r13i1p2f1, r14i1p1f1, r14i1p2f1, r15i1p1f1, r15i1p2f1, r16i1p1f1, r16i1p2f1, r17i1p1f1, r17i1p2f1, r18i1p1f1, r18i1p2f1, r19i1p1f1, r19i1p2f1 |
| CESM2 | r1i1p1f1, r2i1p1f1 | — | — | — | r1i1p1f1 | r10i1p1f1, r11i1p1f1, r1i1p1f1, r2i1p1f1, r3i1p1f1, r4i1p1f1, r5i1p1f1, r6i1p1f1, r7i1p1f1, r8i1p1f1, r9i1p1f1 |
| CESM2-WACCAM | r1i1p1f1 | — | — | — | r1i1p1f1 | r1i1p1f1, r2i1p1f1, r3i1p1f1 |
| CNRM-CM6-1 | r1i1p1f2, r2i1p1f2, r3i1p1f2, r4i1p1f2, r5i1p1f2, r6i1p1f2 | — | — | — | r1i1p1f2 | r1i1p1f2, r2i1p1f2, r3i1p1f2, r4i1p1f2, r5i1p1f2, r6i1p1f2, r7i1p1f2, r8i1p1f2, r9i1p1f2, r10i1p1f2, r11i1p1f2, r12i1p1f2 |
| CNRM-ESM2-1 | r1i1p1f2, r2i1p1f2, r3i1p1f2, r4i1p1f2, r5i1p1f2 | — | — | — | r1i1p1f2 | r1i1p1f2, r2i1p1f2, r3i1p1f2, r4i1p1f2, r5i1p1f2 |
| EC-Earth3 | r1i1p1f1, r6i1p1f1, r9i1p1f1, r11i1p1f1, r13i1p1f1, r15i1p1f1 | — | — | — | r1i1p1f1 | r1i1p1f1, r21i1p1f1, r22i1p1f1, r24i1p1f1, r6i1p1f1, r9i1p1f1, r11i1p1f1, r13i1p1f1, r15i1p1f1 |
| EC-Earth3-Veg | r1i1p1f1, r2i1p1f1 | — | — | — | r1i1p1f1 | r1i1p1f1, r2i1p1f1, r3i1p1f1, r4i1p1f1 |
| GISS-E2-1-G | — | — | — | — | r1i1p1f1 | r1i1p1f1, r2i1p1f1, r3i1p1f1, r4i1p1f1, r5i1p1f1, r6i1p1f1, r7i1p1f1, r8i1p1f1, r9i1p1f1, r10i1p1f1 |
| GISS-E2-1-H | — | — | — | — | r1i1p1f1 | r1i1p1f1, r2i1p1f1, r3i1p1f1, r4i1p1f1, r5i1p1f1, r6i1p1f1, r7i1p1f1, r8i1p1f1, r9i1p1f1, r10i1p1f1 |
| HadGEM3-GC31-LL | — | r1i1p1f3, r2i1p1f3, r3i1p1f3, r4i1p1f3 | r2i1p1f3, r4i1p1f3 | r1i1p1f3, r2i1p1f3, r3i1p1f3, r4i1p1f3 | r1i1p1f1 | r1i1p1f3, r2i1p1f3, r3i1p1f3, r4i1p1f3 |
| IPSL-CM6A-LR | r1i1p1f1 | r1i1p1f1, r2i1p1f1, r3i1p1f1, r4i1p1f1, r5i1p1f1, r6i1p1f1, r7i1p1f1, r8i1p1f1, r9i1p1f1, r10i1p1f1 | r1i1p1f1, r2i1p1f1, r3i1p1f1, r4i1p1f1, r5i1p1f1, r6i1p1f1, r7i1p1f1, r8i1p1f1, r9i1p1f1, r10i1p1f1 | r1i1p1f1,  r2i1p1f1,  r3i1p1f1,  r4i1p1f1,  r5i1p1f1,  r6i1p1f1,  r7i1p1f1,  r8i1p1f1,  r9i1p1f1,  r10i1p1f1 | r1i1p1f1, r1i2p1f1 | r1i1p1f1, r2i1p1f1, r3i1p1f1, r4i1p1f1, r5i1p1f1, r6i1p1f1, r7i1p1f1, r8i1p1f1, r9i1p1f1, r10i1p1f1, r11i1p1f1, r12i1p1f1, r13i1p1f1, r14i1p1f1, r15i1p1f1, r16i1p1f1, r17i1p1f1, r18i1p1f1, r19i1p1f1, r20i1p1f1, r21i1p1f1, r22i1p1f1, r23i1p1f1, r24i1p1f1, r25i1p1f1, r26i1p1f1, r27i1p1f1, r28i1p1f1, r29i1p1f1, r30i1p1f1, r31i1p1f1, r32i1p1f1 |
| MIROC6 | r1i1p1f1, r2i1p1f1, r3i1p1f1 | r1i1p1f1, r2i1p1f1, r3i1p1f1 | r1i1p1f1, r2i1p1f1, r3i1p1f1 | — | r1i1p1f1 | r1i1p1f1, r2i1p1f1, r3i1p1f1, r4i1p1f1, r5i1p1f1, r6i1p1f1, r7i1p1f1, r8i1p1f1, r9i1p1f1, r10i1p1f1 |
| MPI-ESM1-2-HR | r1i1p1f1 | — | — | — | — | r1i1p1f1 |
| NorESM2-LM | — | — | — | — | r1i1p1f1 | r1i1p1f1 |
| SAM0-UNICON | — | — | — | — | r1i1p1f1 | r1i1p1f1 |
| UKESM1-0-LL | r1i1p1f2, r2i1p1f2, r3i1p1f2, r4i1p1f2, r8i1p1f2 | — | — | — | r1i1p1f2 | r1i1p1f2, r2i1p1f2, r3i1p1f2, r4i1p1f2, r5i1p1f3, r6i1p1f3, r7i1p1f3, r8i1p1f2, r9i1p1f2 |

Table 4. Detection times at which the pseudo-observed signals on the ALL fingerprint using the same statistical distribution (GMM) but alternative timescales (1-month and 6-month), and the same timescale (3-month) but alternative statistical distribution (Weibull) became significant at the 95% confidence level.

| Month | 0–10 cm | | | 0–100 cm | | |
| --- | --- | --- | --- | --- | --- | --- |
|  | GMM, 1-month | GMM, 6-month | Weibull, 3-month | GMM, 1-month | GMM, 6-month | Weibull, 3-month |
| Jan | — | 2016 | — | 2014 | 2013 | 2011 |
| Feb | — | — | — | 2014 | 2013 | 2011 |
| Mar | — | — | — | 2009 | 2013 | 2011 |
| Apr | — | — | — | 2001 | 2013 | 2009 |
| May | — | — | — | — | — | 2012 |
| Jun | — | — | — | — | — | — |
| Jul | 2012 | — | — | — | — | — |
| Aug | — | — | 2015 | 2003 | — | — |
| Sept | 2003 | — | 2003 | 1996 | — | — |
| Oct | 2012 | 2012 | 1995 | 1994 | — | 1995 |
| Nov | — | 2005 | 2005 | 1994 | 2015 | 2000 |
| Dec | — | 2005 | — | 2012 | 1997 | 1998 |

Table 5. Detection times at which the pseudo-observed signals on the ALL fingerprint using individual soil moisture products (Mean-ORS, OLC-ORS, EC-ORS) became significant at the 95% confidence level. Em dashes (—) indicate no significant signals. The SSIs were calculated based on the GMM distribution using the 3-month timescale.

| Month | 0–10 cm | | | | 0–100 cm | | | |
| --- | --- | --- | --- | --- | --- | --- | --- | --- |
|  | Mean-ORS | OLC-ORS | EC-ORS | Mean products | Mean-ORS | OLC-ORS | EC-ORS | Mean products |
| Jan | — | — | — | — | 2013 | 2008 | 2014 | 2003 |
| Feb | — | — | — | — | 2014 | 2013 | 2014 | 2006 |
| Mar | — | — | — | 2016 | 2014 | 2013 | 2013 | 2006 |
| Apr | — | — | — | — | 2013 | 2008 | 2008 | 2003 |
| May | — | — | — | — | — | — | — | 2000 |
| Jun | — | — | — | — | — | — | — | — |
| Jul | — | 2011 | — | — | — | — | — | — |
| Aug | — | 2002 | — | 1995 | — | — | — | 2002 |
| Sept | 2005 | 1995 | 2012 | 1995 | — | 2003 | — | 1995 |
| Oct | 2005 | 1995 | 2005 | 1996 | 1994 | 1997 | 1996 | 1996 |
| Nov | 2012 | 1995 | — | 1994 | 1997 | 2000 | 1997 | 2000 |
| Dec | — | 2015 | — | 2012 | 2002 | 2000 | 1994 | 1994 |

Table 6. Detection times at which the pseudo-observed signals on the ALL fingerprint using various statistical distributions (GMM, Weibull) and timescales (1-, 3-, and 6-month) became significant at the 95% confidence level. The calculation of the fingerprints, noises, and signals only used the CMIP6 models and ensemble members that have both piControl and ALL simulations. Em dashes (—) indicate no significant signals.

| Month | 0–10 cm | | | | 0–100 cm | | | |
| --- | --- | --- | --- | --- | --- | --- | --- | --- |
|  | GMM, 1-month | GMM, 3-month | GMM, 6-month | Weibull, 3-month | GMM, 1-month | GMM, 3-month | GMM, 6-month | Weibull, 3-month |
| Jan | — | — | — | — | — | 2014 | 2013 | 2011 |
| Feb | — | — | — | — | 2016 | 2015 | 2013 | 2013 |
| Mar | — | — | — | — | 2013 | 2015 | 2013 | 2013 |
| Apr | — | — | — | — | 2007 | 2013 | 2013 | 2011 |
| May | — | — | — | — | — | — | — | — |
| Jun | — | — | — | — | — | — | — | — |
| Jul | — | — | — | — | — | — | — | — |
| Aug | 2002 | 2015 | — | — | 2003 | — | — | — |
| Sept | 2005 | 2002 | — | 2003 | 1994 | 2006 | — | 2006 |
| Oct | 2012 | 1995 | 2015 | 1995 | 1994 | 1994 | — | 1995 |
| Nov | — | 2005 | 2005 | 2007 | 1994 | 1994 | 2015 | 1995 |
| Dec | — | — | 2012 | — | 2012 | 1994 | 1994 | 1998 |

Table 7. Same as Table 5, but the calculation of the fingerprints, noises, and signals only used the CMIP6 models and ensemble members that have both piControl and ALL simulations.

| Month | 0–10 cm | | | | 0–100 cm | | | |
| --- | --- | --- | --- | --- | --- | --- | --- | --- |
|  | Mean-ORS | OLC-ORS | EC-ORS | Mean products | Mean-ORS | OLC-ORS | EC-ORS | Mean products |
| Jan | — | — | — | — | 2015 | 2011 | 2016 | 2003 |
| Feb | — | — | — | — | 2016 | 2014 | 2016 | 2006 |
| Mar | — | — | — | — | 2016 | 2014 | 2015 | 2006 |
| Apr | — | — | — | — | 2016 | 2013 | 2011 | 2002 |
| May | — | — | — | — | — | — | — | 2001 |
| Jun | — | — | — | — | — | — | — | — |
| Jul | — | — | — | — | — | — | — | — |
| Aug | — | 2002 | — | 1995 | — | — | — | 2002 |
| Sept | 2005 | 1995 | 2012 | 1995 | — | 2002 | — | 1994 |
| Oct | 2005 | 1995 | 2005 | 1994 | 1994 | 1994 | 1994 | 1994 |
| Nov | 2015 | 1995 | — | 1994 | 1994 | 1994 | 1994 | 1991 |
| Dec | — | 2015 | — | 2015 | 2002 | 2000 | 1994 | 1994 |

# Supplementary References

1. Wang, Y. *et al.* Development of observation-based global multi-layer soil moisture products for the period 1970-2016. *Earth Syst. Sci. Data* **13**, 4385–4405 (2021).

2. Pinzon, J. E. & Tucker, C. J. A Non-Stationary 1981–2012 AVHRR NDVI3g Time Series. *Remote Sensing* vol. 6 6929–6960 Preprint at https://doi.org/10.3390/rs6086929 (2014).

3. Kim, H. & Lakshmi, V. Global Dynamics of Stored Precipitation Water in the Topsoil Layer From Satellite and Reanalysis Data. *Water Resources Research* **55**, 3328–3346 (2019).

4. Gillett, N. P. *et al.* The Detection and Attribution Model Intercomparison Project (DAMIP v1.0) Contribution to CMIP6. *Geoscientific Model Development* **9**, 3685–3697 (2016).

5. Eyring, V. *et al.* Overview of the Coupled Model Intercomparison Project Phase 6 (CMIP6) Experimental Design and Organization. *Geoscientific Model Development* **9**, 1937–1958 (2016).

6. ESGF. Earth System Grid Federation, Lawrence Livermore National Laboratory. esgf-node.llnl.gov/ (2019).

7. Marvel, K. *et al.* Twentieth-Century Hydroclimate Changes Consistent with Human Influence. *Nature* **569**, 59–65 (2019).

8. Santer, B. D. *et al.* Human Influence on the Seasonal Cycle of Tropospheric Temperature. *Science* **361**, eaas8806 (2018).

9. Santer, B. D. *et al.* Quantifying Stochastic Uncertainty in Detection Time of Human-Caused Climate Signals. *Proceedings of the National Academy of Sciences* **116**, 19821–19827 (2019).

10. Marvel, K. *et al.* External Influences on Modeled and Observed Cloud Trends. *Journal of Climate* **28**, 4820–4840 (2015).

11. Marvel, K. & Bonfils, C. Identifying External Influences on Global Precipitation. *Proceedings of the National Academy of Sciences of the United States of America* **110**, 19301–19306 (2013).

12. Bonfils, C. J. W. *et al.* Human Influence on Joint Changes in Temperature, Rainfall and Continental Aridity. *Nature Climate Change* **10**, 726–731 (2020).

13. Gagné, M.-È., Kirchmeier-Young, M. C., Gillett, N. P. & Fyfe, J. C. Arctic Sea Ice Response to the Eruptions of Agung, El Chichón, and Pinatubo. *Journal of Geophysical Research: Atmospheres* **122**, 8071–8078 (2017).

14. AghaKouchak, A. A Baseline Probabilistic Drought Forecasting Framework Using Standardized Soil Moisture Index: Application to the 2012 United States Drought. *Hydrological and Earth System Sciences* **18**, 2485–2492 (2014).

15. Modanesi, S., Massari, C., Camici, S., Brocca, L. & Amarnath, G. Do Satellite Surface Soil Moisture Observations Better Retain Information About Crop-Yield Variability in Drought Conditions? *Water Resources Research* **56**, e2019WR025855 (2020).

16. McKee, T. B., Doesken, N. J. & Kleist, J. The Relationship of Drought Frequency and Duration to Time Scales. in *Eighth Conference on Applied Climatology* 1–6 (1993).

17. Vilasa, L., Miralles, D. G., de Jeu, R. A. M. & Dolman, A. J. Global Soil Moisture Bimodality in Satellite Observations and Climate Models. *Journal of Geophysical Research: Atmospheres* **122**, 4299–4311 (2017).

18. Shukla, S., Steinemann, A. C. & Lettenmaier, D. P. Drought Monitoring for Washington State: Indicators and Applications. *Journal of Hydrometeorology* **12**, 66–83 (2010).

19. Sarojini, B. B., Stott, P. A. & Black, E. Detection and Attribution of Human Influence on Regional Precipitation. *Nature Climate Change* **6**, 669–675 (2016).

20. Zhang, X. *et al.* Detection of Human Influence on Twentieth-Century Precipitation Trends. *Nature* **448**, 461–465 (2007).

21. Douville, H., Ribes, A., Decharme, B., Alkama, R. & Sheffield, J. Anthropogenic Influence on Multidecadal Changes in Reconstructed Global Evapotranspiration. *Nature Climate Change* **3**, 59–62 (2013).

22. Storch, H. von & Zwiers, F. W. *Statistical Analysis in Climate Research*. (Cambridge University Press, 1999). doi:10.1017/CBO9780511612336.

23. Tokarska, K. B. *et al.* Past warming trend constrains future warming in CMIP6 models. *Sci. Adv.* **6**, eaaz9549 (2020).

24. Winkler, A. J., Myneni, R. B. & Brovkin, V. Investigating the applicability of emergent constraints. *Earth Syst. Dynam.* **10**, 501–523 (2019).

25. Douville, H. & Plazzotta, M. Midlatitude Summer Drying: An Underestimated Threat in CMIP5 Models? *Geophysical Research Letters* **44**, 9967–9975 (2017).

26. Draper, N. R. & Smith, H. *Applied Regression Analysis, 3rd Edition*. (John Wiley & Sons, Inc., 1998).

27. Harris, I., Osborn, T. J., Jones, P. & Lister, D. Version 4 of the CRU TS monthly high-resolution gridded multivariate climate dataset. *Sci. Data* **7**, 109 (2020).

28. Chen, M., Xie, P., Janowiak, J. E. & Arkin, P. A. Global land precipitation: A 50-yr monthly analysis based on gauge observations. *J. Hydrometeorol.* **3**, 18 (2002).

29. Willmott, C. J. & Matsuura, K. Terrestrial air temperature and precipitation: Monthly and annual time series (1950-1999). http://climate.geog.udel.edu/~climate/html_pages/README.ghcn_ts2.html (2001).

30. Schneider, U. *et al.* GPCC full data reanalysis version 6.0 at 0.5°: Monthly land-surface precipitation from rain-gauges built on GTS-based and historic data. (2011) doi:10.5676/DWD_GPCC/FD_M_V7_050.

31. Yang, Y., Roderick, M. L., Zhang, S., McVicar, T. R. & Donohue, R. J. Hydrologic implications of vegetation response to elevated CO_2_ in climate projections. *Nature Clim Change* **9**, 44–48 (2019).

32. Compo, G. P., Whitaker, J. S. & Sardeshmukh, P. D. Feasibility of a 100-year reanalysis using only surface pressure data. *Bull. Amer. Meteor. Soc.* **87**, 175–190 (2006).

33. C3S. ERA5: Fifth Generation of ECMWF Atmospheric Reanalyses of the Global Climate. *Copernicus Climate Change Service Climate Data Store* https://cds.climate.copernicus.eu/cdsapp#!/home (2017).

34. Beaudoing, H. K., Rodell, M. & NASA/GSFC/HSL. GLDAS Noah Land Surface Model L4 Monthly 0.25 x 0.25 Degree V2.0. (2015) doi:10.5067/9SQ1B3ZXP2C5.

35. Hoerl, A. E. & Kennard, R. W. Ridge regression: Biased estimation for nonorthogonal problems. *Technometrics* **12**, 55–67 (1970).

36. Huntzinger, D. N. *et al.* NACP MsTMIP: Global 0.5-degree Model Outputs in Standard Format, Version 1.0. Preprint at https://doi.org/10.3334/ornldaac/1225 (2018).

37. Sitch, S. *et al.* Evaluation of the Terrestrial Carbon Cycle, Future Plant Geography and Climate-Carbon Cycle Feedbacks Using Five Dynamic Global Vegetation Models (DGVMs). *Global Change Biology* **14**, 2015–2039 (2008).

38. Martens, B. *et al.* GLEAM v3: Satellite-Based Land Evaporation and Root-Zone Soil Moisture. *Geoscientific Model Development* **10**, 1903–1925 (2017).

39. Miralles, D. G. *et al.* Global Land-Surface Evaporation Estimated from Satellite-Based Observations. *Hydrology and Earth System Sciences* **15**, 453–469 (2011).

40. Laloyaux, P., De Boisséson, E. & Dahlgren, P. CERA-20C : An Earth System Approach to Climate Reanalysis. *ECMWF Newsletter* **150**, 25–30 (2017).

41. Poli, P. *et al.* ERA-20C: An Atmospheric Reanalysis of the Twentieth Century. *Journal of Climate* **29**, 4083–4097 (2016).

42. Dee, D. P. *et al.* The ERA-Interim Reanalysis: Configuration and Performance of the Data Assimilation System. *Quarterly Journal of the Royal Meteorological Society* **137**, 553–597 (2011).

43. Balsamo, G. *et al.* ERA-Interim/Land: A Global Land Surface Reanalysis Data Set. *Hydrology and Earth System Sciences* **19**, 389–407 (2015).

44. Rodell, M. *et al.* The Global Land Data Assimilation System. *Bulletin of the American Meteorological Society* **85**, 381–394 (2004).

45. Dorigo, W. *et al.* ESA CCI Soil Moisture for Improved Earth System Understanding: State-of-The Art and Future Directions. *Remote Sensing of Environment* **203**, 185–215 (2017).

46. Hobeichi, S., Abramowitz, G., Evans, J. & Ukkola, A. Derived Optimal Linear Combination Evapotranspiration (DOLCE): A Global Gridded Synthesis Estimate. *Hydrology and Earth System Sciences* **22**, 1317–1336 (2018).

47. Hobeichi, S., Abramowitz, G., Evans, J. & Beck, H. E. Linear Optimal Runoff Aggregate (LORA): A Global Gridded Synthesis Runoff Product. *Hydrology and Earth System Sciences* **23**, 851–870 (2019).

48. Dorigo, W. A. *et al.* The International Soil Moisture Network: A Data Hosting Facility for Global in situ Soil Moisture Measurements. *Hydrology and Earth System Sciences* **15**, 1675–1698 (2011).

49. Mystakidis, S., Davin, E. L., Gruber, N. & Seneviratne, S. I. Constraining Future Terrestrial Carbon Cycle Projections Using Observation-Based Water and Carbon Flux Estimates. *Global Change Biology* **22**, 2198–2215 (2016).

50. Harris, I., Jones, P. D., Osborn, T. J. & Lister, D. H. Updated High-Resolution Grids of Monthly Climatic Observations – the CRU TS3.10 Dataset. *International Journal of Climatology* **34**, 623–642 (2014).

51. Taylor, K., Stouffer, R. & Meehl, G. A Summary of the CMIP5 Experiment Design. *World Climate Research Programme* 1–33 Preprint at (2011).

1. † This manuscript has been authored by UT-Battelle LLC under Contract No. DE-AC05-00OR22725 with the US Department of Energy (DOE). The US government retains and the publisher, by accepting the article for publication, acknowledges that the US government retains a nonexclusive, paid-up, irrevocable, worldwide license to publish or reproduce the published form of this manuscript, or allow others to do so, for US government purposes. DOE will provide public access to these results of federally sponsored research in accordance with the DOE Public Access Plan (<http://energy.gov/downloads/doe-public-access-plan>). [↑](#footnote-ref-2)
